# Biological activity of Pt^IV^ prodrugs triggered by riboflavin-mediated bioorthogonal photocatalysis

**DOI:** 10.1038/s41598-018-35655-2

**Published:** 2018-11-21

**Authors:** Silvia Alonso-de Castro, Alessio Terenzi, Sonja Hager, Bernhard Englinger, Adriana Faraone, Javier Calvo Martínez, Mathea Sophia Galanski, Bernhard K. Keppler, Walter Berger, Luca Salassa

**Affiliations:** 1 CIC biomaGUNE, Paseo de Miramón 182, Donostia, 20014 Spain; 2 Donostia International Physics Center, Paseo Manuel de Lardizabal 4, Donostia, 20018 Spain; 3 Institute of Inorganic Chemistry, University of Vienna, Waehringerstrasse 42, A-1090 Vienna, Austria; 4 Research Platform “Translational Cancer Therapy Research”, University of Vienna and Medical University of Vienna, A-1090 Vienna, Austria; 5 Institute of Cancer Research and Comprehensive Cancer Center, Medical University of Vienna, Borschkegasse 8a, A-1090 Vienna, Austria; 6 Ikerbasque, Basque Foundation for Science, Bilbao, 48011 Spain

**Keywords:** Cyclobutanedicarboxylate, Prodrug System, Cell Viability Experiments, Free Cisplatin, Photodynamic Therapy, Chemical biology, Coordination chemistry

## Abstract

We have recently demonstrated that riboflavin (**Rf**) functions as unconventional bioorthogonal photocatalyst for the activation of Pt^IV^ prodrugs. In this study, we show how the combination of light and **Rf** with two Pt^IV^ prodrugs is a feasible strategy for light-mediated pancreatic cancer cell death induction. In Capan-1 cells, which have high tolerance against photodynamic therapy, **Rf**-mediated activation of the cisplatin and carboplatin prodrugs *cis*,*cis*,*trans*-[Pt(NH_3_)_2_(Cl)_2_(O_2_CCH_2_CH_2_CO_2_H)_2_] (**1**) and *cis*,*cis*,*trans*-[Pt(NH_3_)_2_(CBDCA)(O_2_CCH_2_CH_2_CO_2_H)_2_] (**2**, where CBDCA = cyclobutane dicarboxylate) resulted in pronounced reduction of the cell viability, including under hypoxia conditions. Such photoactivation mode occurs to a considerable extent intracellularly, as demonstrated for **1** by uptake and cell viability experiments. ^195^Pt NMR, DNA binding studies using circular dichroism, mass spectrometry and immunofluorescence microscopy were performed using the **Rf**-**1** catalyst-substrate pair and indicated that cell death is associated with the efficient light-induced formation of cisplatin. Accordingly, Western blot analysis revealed signs of DNA damage and activation of cell death pathways through **Rf**-mediated photochemical activation. Phosphorylation of H_2_AX as indicator for DNA damage, was detected for **Rf-1** in a strictly light-dependent fashion while in case of free cisplatin also in the dark. Photochemical induction of nuclear pH_2_AX foci by **Rf-1** was confirmed in fluorescence microscopy again proving efficient light-induced cisplatin release from the prodrug system.

## Introduction

Riboflavin (**Rf**), vitamin B2, is an essential exogenous biomolecule for our metabolism. **Rf** is rapidly converted intracellularly into flavin mononucleotide (FMN) and flavin adenine dinucleotide (**FAD**), which are then incorporated into a multitude of flavoproteins and flavoenzymes. These have motivated an enormous interest in research due to their key role in biological systems where they catalyze the oxidation of a broad range of substrates (sugars, alcohols or aminoacids), participate in oxidation/reduction processes involved in detoxification, and play a photocatalytic role in DNA repair^1,2^.

**Rf** has outstanding photophysical and photochemical features that make this natural photosensitizer a good candidate for light-triggered applications in medicine. A noteworthy use of **Rf** is the Mirasol PRT™ process employed to treat blood for transfusion, in which **Rf** inactivates significant levels of viruses and bacteria in platelets and plasma upon UV light irradiation^3^. Besides, **Rf** has been investigated for application in photodynamic therapy (PDT). This clinically approved treatment for cancer and other diseases employs light to activate a photosensitizer and generate singlet oxygen and other reactive oxygen species (ROS) capable to induce cell death locally in irradiated areas^4^. Under light irradiation, **Rf** generates singlet oxygen in aerated solutions with higher quantum yields (Φ_Δ_ = 0.54 ± 0.07) than other exogenous photosensitizers used in the clinics, such as Photofrin^5^.

A current limitation in PDT is the absolute O_2_-dependency, which reduces the effectiveness of this treatment in hypoxic tissues^4^. The search for newer O_2_-independent strategies with improved photosensitizers is an emerging focus in PDT and transition metal complexes have raised significant interest as photoactivatable prodrug candidates^6^. Despite their encouraging profiles as antineoplastic agents, light-switchable metal complexes, and in particular Pt^IV^ prodrugs, often suffer from modest absorption properties. For this reason, innovative approaches have been explored to turn on their activity at more convenient wavelengths^7–9^.

**Figure 1:**
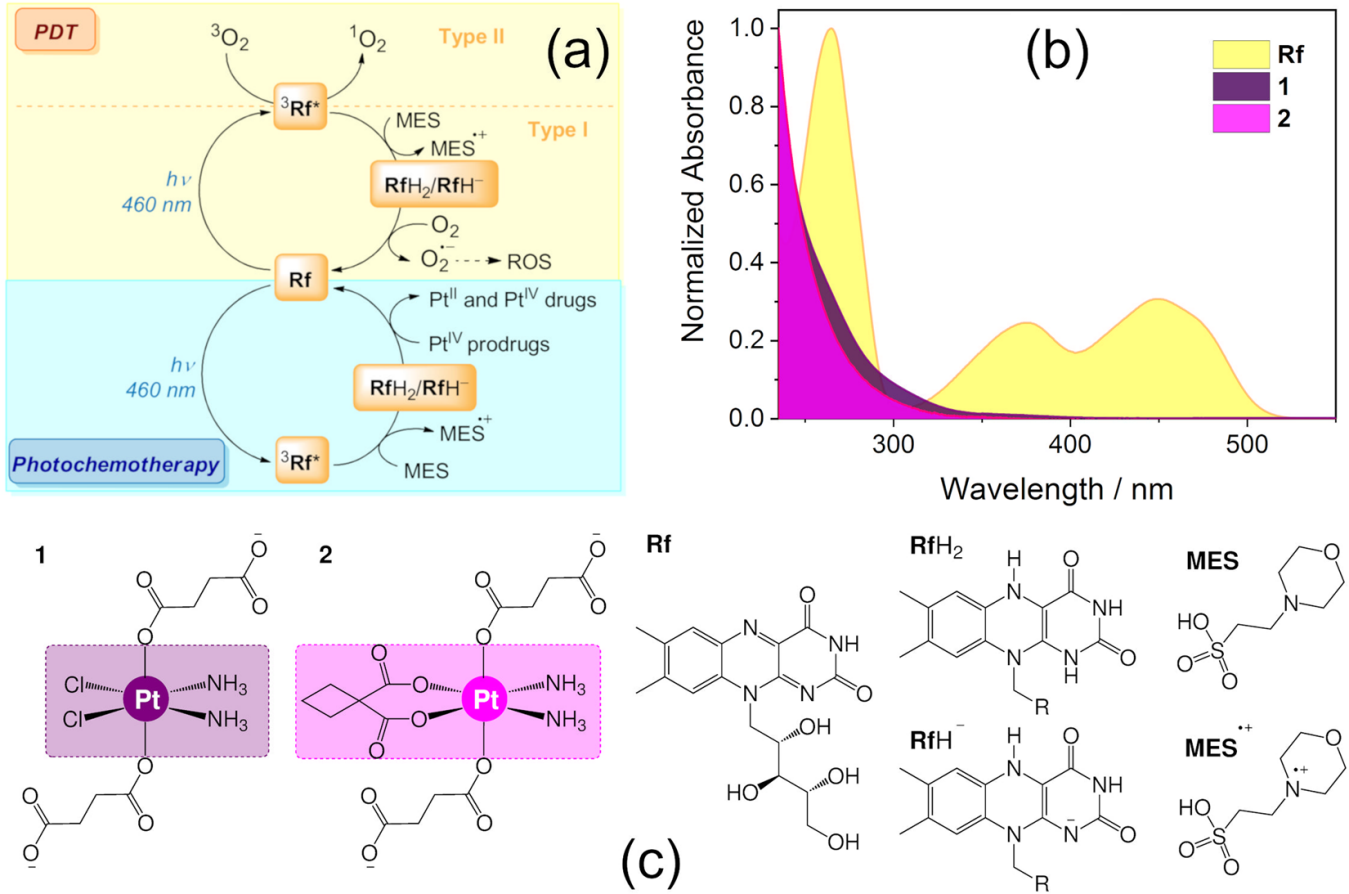
(**a**) Proposed photocatalytic mechanism for the bioorthogonal activation of Pt^IV^ prodrugs by **Rf** (and other flavins); (**b**) UV-Vis absorption spectra of **Rf**, **1** and **2**; and (**c**) Schematic structures of the Pt^IV^ prodrugs employed in this study (**1** and **2**), and chemical species involved in their photocatalytic activation.

In our search for novel activation strategies, we recently showed that **Rf** and other flavins, including flavoproteins, can simultaneously act as photosensitizers and unconventional photocatalysts for the selective activation of anticancer metal-based prodrug candidates such as Pt^IV^ complexes. These photocatalytic reactions efficiently deliver active Pt^II^ species and take place by means of blue rather than UV-A light in the presence of zwitterionic buffers such as MES (2-(N-morpholino)ethanesulfonic acid), as well as with biological electron donors such as NAD(P)H^10,11^. Importantly, low doses of blue light are sufficient to fully convert high concentrations of Pt^IV^ prodrugs in buffer solution and in cell culture medium. The **Rf** selectivity for Pt^IV^ complexes demonstrated in the biological environment defines the bioorthogonal nature of these photocatalytic reactions^10,11^. According to our current understanding of the process (Fig. 1)^10^, light excitation of **Rf** at 460 nm results in the formation of its triplet excited state, which is a highly oxidant species able to extract electrons from sacrificial electron donors (e.g. MES) to give the reduced riboflavin forms **Rf**H_2_ or **Rf**H^–^. Afterwards, **Rf**H_2_ or **Rf**H^–^ catalytically affords the conversion of Pt^IV^ complexes into biologically active Pt^II^ species.

This report provides new insights on the cytotoxic effect and mechanism of action of two Pt^IV^ prodrugs, namely *cis*,*cis*,*trans*-[Pt(NH_3_)_2_(Cl)_2_(O_2_CCH_2_CH_2_CO_2_H)_2_] (**1**) and *cis*,*cis*,*trans*-[Pt(NH_3_)_2_(CBDCA)(O_2_CCH_2_CH_2_CO_2_H)_2_] (**2**, where CBDCA = cyclobutane dicarboxylate), upon flavin-mediated photocatalytic activation. In particular, we describe here how the cisplatin prodrug **1** can be used in combination with **Rf** and extremely low doses of blue light to eradicate human pancreatic adenocarcinoma cells (Capan-1). This cell model was chosen because of its relatively high tolerance against PDT which allows establishing the interplay between this type of treatment and photoactivatable Pt^IV^ prodrugs.

Pancreatic cancer is the 4^th^ leading cause of cancer-related deaths, and its 5-years survival rate is lower than 5%. Recent studies reveal alternative treatments may change this dreadful prognosis^12–14^. For example, a prospective chemotherapeutic option consists on the combinatory treatment of gemcitabine with cisplatin, which revealed a significant improvement in the 6-months survival rate compared to the administration of gemcitabine alone^14^. In the treatment of pancreatic cancer, PDT also receives special attention since it is not accompanied by the heavy side effects of systemic chemotherapy^15^. Moreover, both platinum drugs^16^ and PDT^17^ have been shown to activate immune responses against pancreatic tumors and even immunogenic cell death^18,19^.

The photochemistry of **Rf** may be a new tool to integrate singlet oxygen sensitization and bioorthogonal photocatalysis towards Pt^IV^ prodrugs for exploiting some of the advantages introduced by PDT and combination therapies in the treatment of pancreatic cancer. A combined strategy for PDT and platinum drug activation may lead to synergistic antitumor drug and immune responses localized in the malignant tissue^14^.

## Results and Discussion

### Antiproliferative properties of Pt^IV^ complexes photocatalytically activated by riboflavin

The potential of **Rf** in PDT is well documented^15–17^. We reasoned that our bioorthogonal photocatalytic strategy would be a worthy strategy to test in those cancers in which PDT is of limited efficiency or might become ineffective because of oxygen consumption^20^. Hence, we initially screened **Rf** photocytotoxicity in a number of human cell lines derived from different tumors including colon (SW480 and HTC116), ovarian (A2780 and A2780cis), cervix (HeLa –derivative KB-3-1), and pancreatic (Capan-1) carcinomas as well as a melanoma (VM47), and glioblastoma (H35 and H52).

In this preliminary screen (Fig. S1), cells were treated with **Rf** concentrations up to 20 µM. MES (1% vol., 2 mM) was added to the culture medium to prevent **Rf** photodecomposition^1^ and mimic the conditions used for the catalytic activation of Pt^IV^ prodrugs (*vide infra*). It is worth pointing out that no toxicity was observed due to the addition of MES alone (Fig. S2).

Cell viability experiments consisted in a drug-to-light interval of 1 h following administration, 1 min of blue light irradiation (460 nm, 0.36 J·cm^–2^) and 6 h of incubation. Afterwards, media was renewed and cells were incubated for a total of 72 h in the dark. Non-irradiated controls were directly incubated 7 h in the dark and then handled as described above for light-activated samples. After the 72 h, cell viability was determined by the MTT assay.

Among the panel of cell lines tested, Capan–1 showed the greatest survival and resistance against the **Rf** photosensitizing effects, retaining > 75% viability at 25 µM **Rf** under light exposure. The low sensitivity of Capan-1 cells to PDT is enigmatic but can be ascribed to the high levels of antioxidant molecules (such as glutathione) and enzymes (such as superoxide dismutase, catalase and glutathione peroxidase) present in this cell line^4^. Moreover, Capan-1 cells harbor a mutated p53 tumor suppressor causing general cell death resistance^20^ but also a BRCA2 mutation leading to enhanced cisplatin sensitivity^21^. Conversely^22^, all the other cell lines displayed a cell viability below 10% at concentrations ranging between 5 and 15 µM **Rf** (Fig. S1).

In light of the capacity of Capan-1 cells to endure PDT at the light dose employed, we extended the concentration range of **Rf** and studied how the co-administration of MES (1% vol., 2 mM) affected **Rf** phototoxicity. In the absence of MES, no toxicity was observed for concentrations of **Rf** up to 75 µM, both in dark and under light irradiation. However, addition of MES favors **Rf** phototoxic action, significantly decreasing cell survival (40% cell death at 75 µM **Rf**, Fig. S3). On the basis of our previous work, this finding was anticipated. MES, as well as other zwitterionic buffers, preserves **Rf** from photodecomposition acting as electron donor, hence, extending the generation of singlet oxygen and other ROS^10^. In an analogous manner, acetylation of ribityl chain was demonstrated to improve **Rf** photostability and the PDT capacity of this photosensitizer^17^.

For sake of comparison, we also tested **FAD** under similar experimental conditions, observing a less pronounced but comparable behavior to **Rf**, and confirming the key role of MES as PDT enhancing agent for flavin photosensitizers (Fig. S4).

Taking into consideration these results, we selected Capan-1 cells to evaluate the bioorthogonal **Rf**-mediated photocatalytic activation of two Pt^IV^ prodrug candidates (Fig. 1). The compounds investigated were *cis*,*cis*,*trans*-[Pt(NH_3_)_2_(Cl)_2_(O_2_CCH_2_CH_2_CO_2_H)_2_] (**1**) and *cis*,*cis*,*trans*-[Pt(NH_3_)_2_(CBDCA)(O_2_CCH_2_CH_2_CO_2_H)_2_] (**2**), which are deprotonated at carboxylic groups at physiological pH^10^, and are prodrugs of cisplatin and carboplatin respectively. Only a 10% load of the **Rf** catalyst was used in this set of experiments.

**Figure 2:**
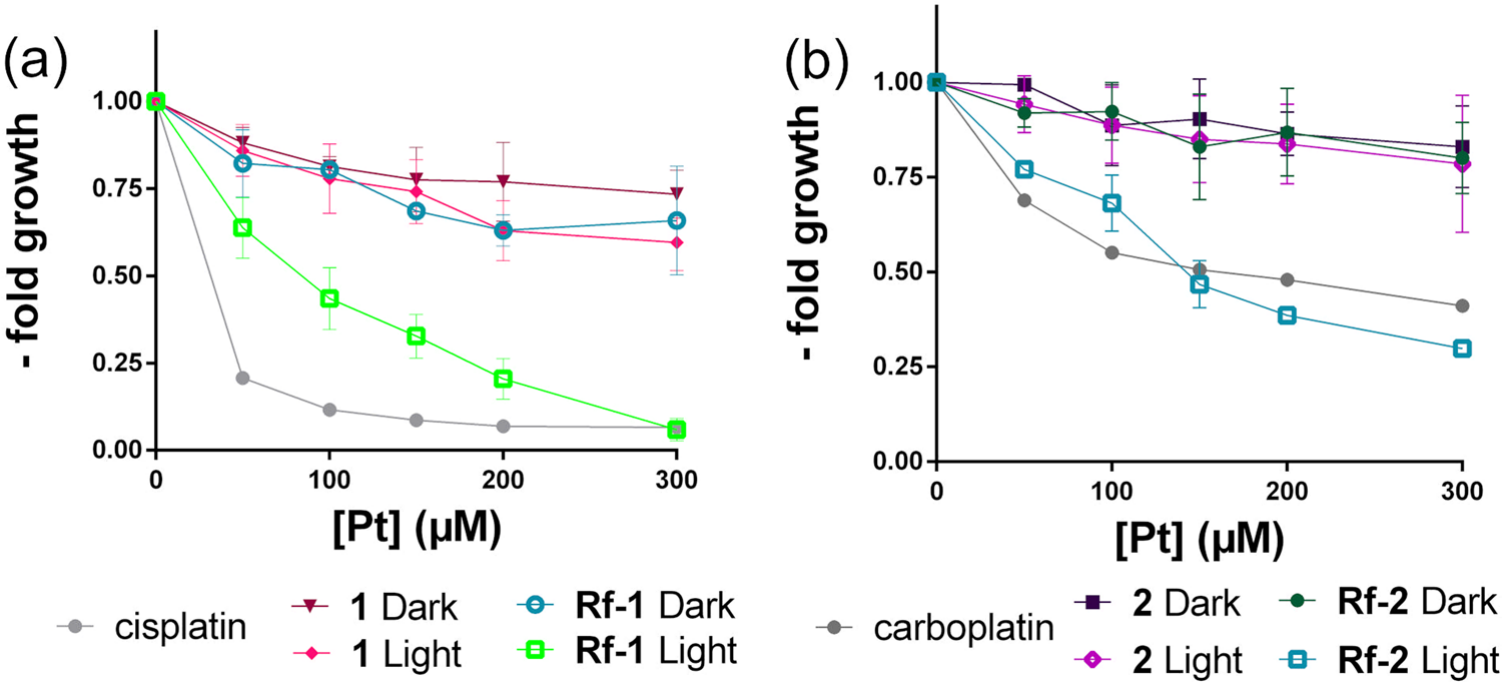
Photocatalytic effect of **Rf**-**1** and **Rf**-**2** against Capan-1 cells. Cell viability following exposure to (**a**) **1** and **Rf**-**1** and (**b**) **2** and **Rf**-**2** in the dark and under light irradiation (460 nm, 0.36 J·cm^–2^) compared to dark controls and cisplatin (dark).

Both **Rf**-**1** and **Rf**-**2** catalyst-substrate pairs showed improved photocytotoxic profiles with respect to **Rf** alone under a light dose of only 0.36 J·cm^–2^. In fact, **Rf**-**1** and **Rf**-**2** induced reductions in cell viability analogous to the direct administration of the corresponding Pt^II^ drugs (Fig. 2). At low Pt^IV^ concentrations, **Rf**-**1** is less effective in inducing cell death than cisplatin whereas light-irradiated **Rf**-**2** and carboplatin had similar cytotoxicity profiles. As shown previously by the Keppler group^23^ and us^24^, this result may be ascribed to the fact that (photo)reductive elimination reactions of Pt^IV^ complexes are more likely to lead to single Pt^II^ products in the case of carboplatin derivatives rather than for cisplatin ones. Control samples, which included **1** and **2** under light irradiation and in the dark, and **Rf**-**1**/**2** in the dark, showed minimal toxicity. The Pt-induced phototoxicity was lower for both **Rf**-**1** and **Rf**-**2** in the absence of MES (Figs S5 and S6). When employed as photocatalyst, **FAD** affected cell viability in a less efficient manner compared to **Rf** and even less without MES (Figs S7–10).

Motivated by the activity of **Rf**-**1** upon light irradiation, we investigated in Capan-1 the role of catalyst loading and the O_2_-dependency on the prodrug activation. Fixing the concentration of **1** at 150 µM, we modified the catalyst-substrate ratio using 15 and 75 µM **Rf** (Fig. S11). **Rf**-**1** showed high antiproliferative capacity and a small difference (10%) in cell viability between the two **Rf**:**1** ratios was found. Such disparity can be assigned to **Rf** phototoxicity at higher concentrations (>25 µM, Fig. S2). Under hypoxic conditions (pO_2_ < 1%), light-irradiated **Rf** alone does not produce any cytotoxicity, meanwhile **Rf**-**1** retains the biological activity exhibited in normoxia. For example, **Rf**-**1** at the catalyst-substrate ratio of 15:150 µM reduced Capan-1 cell viability to ca. 30% in both normoxic and hypoxic conditions (Fig. S12). Therefore, flavin-based photocatalysis towards Pt^IV^ complexes is an O_2_-independent strategy that could function in hypoxic environments where PDT generally does not work and in treatments where PDT has lost effectiveness because of O_2_ depletion.

A remarkable aspect of our strategy is the low light dose needed for the Pt^IV^ prodrug activation. Only 0.36 J·cm^–2^ of 460-nm light was already sufficient to switch on cytotoxicity and provide antiproliferative effects resembling free cisplatin and carboplatin. In the case of analogue Pt^IV^ complexes, the light dose needed for UV-A and blue light activation was more than 15 times higher than for **Rf**-mediated photocatalysis^25^.

### Insights in the mechanism of action

The remarkable light-induced activity of **Rf**-**1** in Capan-1 cells prompt us to select this prodrug system for further investigations of its mechanism of action. Additional experiments included ^195^Pt NMR speciation studies, DNA interaction experiments by circular dichroism spectroscopy (CD) and mass spectrometry, cell uptake by ICP-MS, Western blotting analysis and immunofluorescence microscopy.

^1^H,^195^Pt-HSQC NMR experiments were performed to determine the speciation of **1** upon **Rf**-mediated photocatalytic activation. The photoreduction of Pt^IV^ metal complexes often results in multiple products, including Pt^II^ and Pt^IV^ species^23^. A sample solution of **1** (7.2 mM) and **Rf** (260 µM) in 2 mM MES (pH 6) was irradiated during 10 min (ratio **Rf:1** 1:27) and its ^195^Pt NMR spectrum collected. The concentration of **1** in these experiments was increased compared to cell viability studies in view of the low sensitivity of ^195^Pt NMR. **Rf** concentration was limited to 260 µM by its modest aqueous solubility.

**Figure 3:**
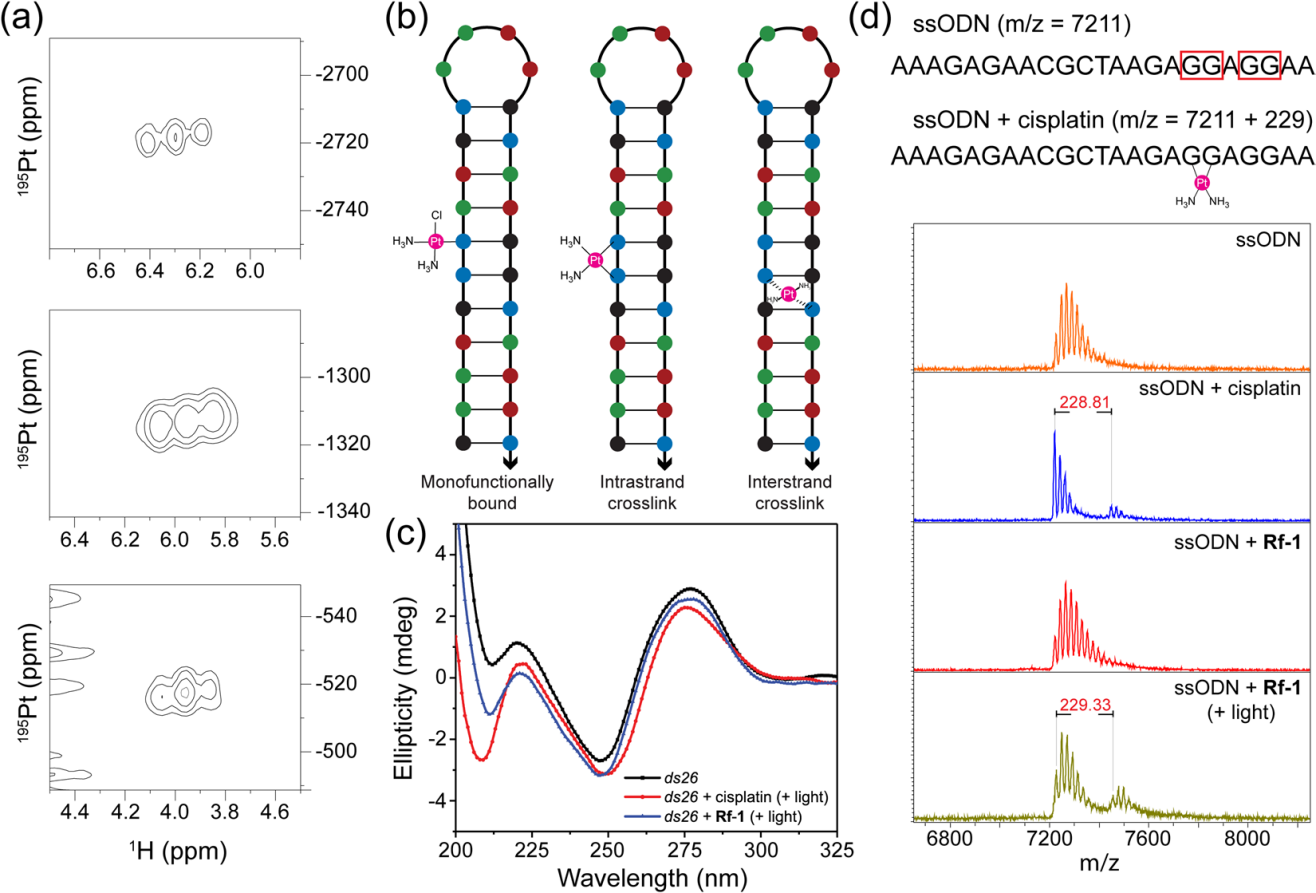
(**a**) ^1^H,^195^Pt-HSQC NMR spectra of light irradiated **Rf**-**1**. Spectra were obtained using 7.2 mM **1** and 267 μM **Rf** (ratio Rf:Pt^IV^ 1:27) in 2 mM MES buffer (pH 6) and irradiating at 460 nm for 10 min. (**b**) Schematic representation of the selected B-DNA model ds26 and the possible Pt-DNA adducts formed upon treatment with cisplatin. (**c**) CD spectra of 1 μM *ds26* with 3 μM cisplatin and **Rf**-**1** (0.3/3 μM) after irradiation and incubation for 48 h at 37 °C. (**d**) MALDI-TOF-MS spectra of 6 μM ssODN with 3 μM cisplatin and **Rf**-**1** (0.3/3 μM) after 1 min irradiation and incubation for 48 h at 37 °C.

^1^H,^195^Pt-HSQC NMR spectroscopy (Fig. 3a) revealed that **1** was not completely converted under the adopted experimental conditions (signal of **1** at around −2720 ppm). Nevertheless, two new Pt species were generated by the photocatalytic process. The signal at −1310 ppm corresponded to an unknown Pt^IV^ species, whose identification remains elusive. Mass spectrometry experiments performed at different irradiation times indicate however the formation of a dimeric Pt species (Fig. S13). Most importantly, the Pt^II^ complex detected at around –520 ppm was identified as cisplatin, demonstrating that the photoreduction of the prodrug indeed took place. Control spectra of cisplatin and **1** with **Rf** (dark) were employed for assignment and are reported in Figs S14–17.

Considering that cisplatin anticancer activity mainly depends on its binding to DNA^26^, we investigated the interaction of the **Rf**-**1** catalyst-prodrug pair with a double stranded DNA model by circular dichroism, a technique used to monitor minor variations on the DNA double helix^27^. We selected the oligodeoxyribonucleotide (ODN) *ds26*, an auto-complementary sequence structured in a hairpin-duplex B-DNA fashion^28^, which is ideal for characterizing the formation of DNA-cisplatin adducts (Fig. 3b) such as: (i) intrastrand crosslinks between neighbouring guanine residues (accounting for 90% of the adducts)^29–31^; (ii) monofunctional adducts; iii) interstrand cross-links^32,33^. CD spectra were collected (Figs 3c and S18) in the dark and after light irradiation (460 nm, 0.36 J·cm^–2^) using MES buffered solutions of cisplatin and **1**. These were incubated with *ds26* for 48 h at 37 °C, either in the presence or absence of **Rf**.

Overall, the global B-conformation of DNA was retained upon complexation with Pt^II^ species. However, spectroscopy data revealed a local perturbation of the DNA structure in the case of DNA treated with light-activated **Rf**-**1** and cisplatin. In particular, platinum species generated upon irradiation of **Rf**-**1** led to a decrease of the positive band of B-DNA at around 275 nm, accompanied by a red shift in the 250–275 nm range. Such spectral change was previously interpreted for cisplatin as a B to C-like DNA conformational change involving the base pairs surrounding the platinated nucleosides. This feature is typical of intrastrand crosslinks which do not disrupt the Watson-Crick hydrogen bonding pattern^31,34,35^. On the other hand, the small increase of the negative band at 250 nm observed for cisplatin and **Rf**-**1** upon light activation ruled out the formation of interstrand crosslinks in *ds26*. This spectroscopic feature might be associated to a non-severe oxidative damage of DNA induced by light, as previously observed by Nowicka *et al*.^36^. In fact, interstrand binding typically leads to a pronounced reduction of this negative CD band as a consequence of the disruption of the hydrogen bonds between the platinated guanines and the complementary cytosines^37^.

Importantly, when compared to controls (Fig. S18), only irradiated **Rf**-**1** presented the same effects as cisplatin. These results further demonstrate the efficacy of our prodrug activation approach to deliver Pt^II^ species capable of binding DNA bases. It is worth pointing out that the impact on the DNA CD bands induced by **Rf**-**1** photoproducts, although following the identical patterns, is less marked. This difference is most probably due to the faster formation of the diaqua active species cis-[Pt(NH_3_)_2_(H_2_O)_2_]^2+^ upon direct addition of cisplatin. On the contrary, **1** can undergo aquation only after photoreduction. Although obtained under different experimental conditions, ^1^H-^195^Pt NMR indicated that photocatalytic activation of **1** can lead to the formation of more than one Pt species, one of which is a Pt^IV^ derivative, supporting a significantly lower DNA binding capacity compared to an equimolar solution of cisplatin.

We further assessed the capacity of the light-activated **Rf**-**1** pair to form DNA-Pt crosslinks in the presence of a properly designed ODN by MALDI mass spectrometry. For this study, we selected the sequence 5′-AAAGAGAACGCTAAGAGGAGGAA-3′, a single stranded ODN (ssODN) incorporating two GG-box motifs. Identical incubation and irradiation conditions as in CD experiments were employed. Figure 3d shows that irradiation of the **Rf**-**1** led to the formation of 1:1 ssODN-Pt adducts with the [Pt(NH_3_)_2_]^2+^ fragment as in the case of cisplatin alone, confirming the production of intrastrand crosslinks upon activation of **1**.

Cellular accumulation is a key aspect in the biological activity of a drug candidate. Therefore, Pt cell uptake experiments in Capan-1 cells were performed under different incubation regimes and irradiation protocols (Fig. S19). The amount of Pt accumulated in cells was quantified using ICP-MS. Cisplatin and **1** alone or in combination with **Rf** (10 μM) were used at a concentration of 100 μM.

The quantity of Pt in Capan-1 cells found after the first hour of incubation in the dark is similar for cisplatin, **1** and **Rf**-**1**. After light was administered, the content of Pt found for **1** and **Rf**-**1** rapidly reached a plateau and did not change further between 3 h and 6 h of additional incubation. Crucially, no significant difference was observed for cells treated with **1** and **Rf**-**1** in the dark and upon light irradiation. On the contrary, cisplatin accumulated to a higher extent over time compared to **1** and **Rf**-**1**, both in the dark and when exposed to light.

The behaviour of light-irradiated **Rf**-**1** suggests that the antiproliferative action of the prodrug complex triggered by **Rf** under our general protocol (1 h preincubation before irradiation) can be principally ascribed to a photocatalytic intracellular activation. In fact, extracellular production of Pt^II^ species would lead to a progressive increase of the Pt content over time, as observed for cisplatin, which is not the case for **1** photocatalytically converted by **Rf**. To confirm this scenario, we performed additional cell viability experiments in which the Pt agents were removed by washing cells with fresh media immediately after 1 h of preincubation and 1 min of light irradiation (Fig. S20). This approach excluded that any toxicity effect could originate from species formed extracellularly. Differently from controls, light-activated **Rf**-**1** under such conditions reduced cell viability in a significant manner confirming the capacity of **Rf** to catalyse intracellularly the formation of cisplatin from **1**. In another control experiment, a higher Pt content was detected in Capan-1 cells in comparison to control samples when **Rf**-**1** was irradiated at 460 nm just after administration and then incubated for overall 7 h. Using such a modified irradiation treatment, the generation of cisplatin occurred completely outside the cell and the viability profile of **Rf**-**1** more closely resembles the one obtained for cisplatin (Figs S19 and S21).

Internalization of **Rf** in cancer cells is extremely rapid (in the order of minutes) due to active and passive transport mechanisms^38^. As demonstrated in previously reported studies, there is a predominance of passive transport over protein-mediated cellular uptake at **Rf** concentrations above normal human plasma levels (ca. 12 nM)^39,40^, which could support co-localization of **1** and **Rf** in the cytosol. Furthermore, the gene expression profile^41^ of Capan-1 cells indicate that this cell line overexpresses riboflavin transporter proteins (solute carrier family 52), hence improving their ability to internalize the vitamin. So, the great majority of the **Rf** photocatalyst is only available within the cell after the first hour of preincubation and its extracellular concentration as well its capacity of generating cisplatin from extracellular **1** should be minimal. This is well in agreement with cell uptake and cytotoxicity results.

### Induction of a light-dependent DNA damage response

**Figure 4:**
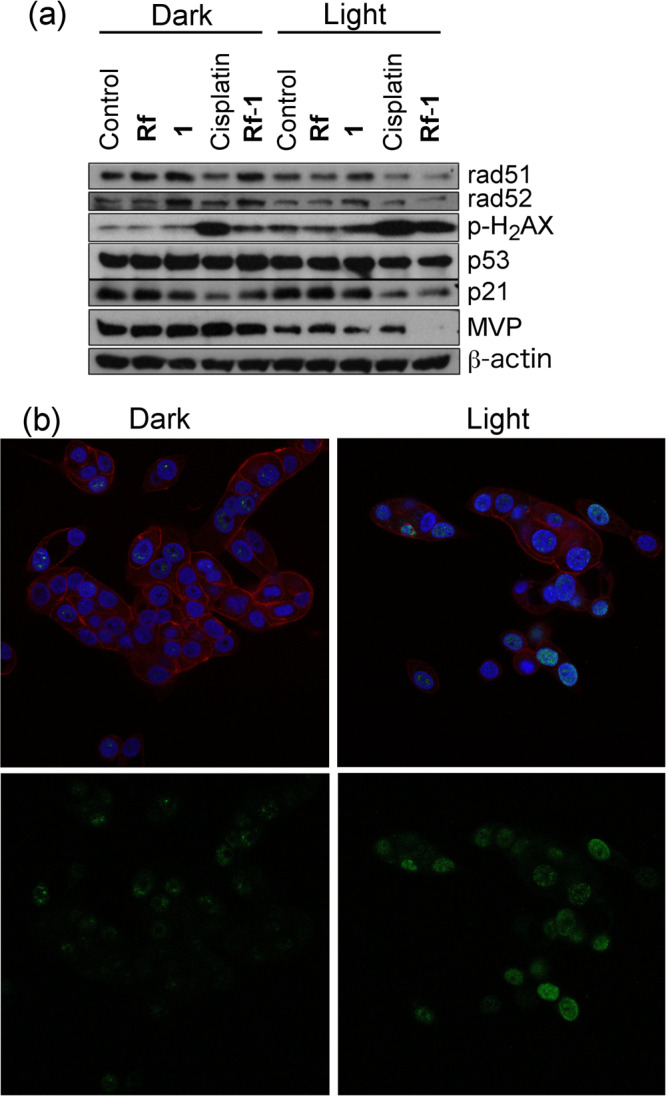
(**a**) Expression/phosphorylation of DNA damage and cell death proteins in Capan-1 cells analysed by Western blotting. Cells were: untreated (lane 1), or treated by 10 µM **Rf** (lane 2), 100 µM **1** (lane 3), 100 µM cisplatin (lane 4) and **Rf**-**1** 10:100 µM (lane 5) in the dark. Same treatments from lane 6 to 10 but under 460-nm light irradiation (460 nm, 0.36 J·cm^–2^). Membranes were probed for the indicated proteins or phosphorylation-specific epitopes. Full-length blots/gels are presented in Supplementary Fig. S22. (**b**) Immunofluorescence microscopy images of the DNA damage marker p-H_2_AX induced by **Rf**-**1** (10:100) in the dark and under 460-nm light irradiation. Treated and fixed Capan-1 cells were stained with DAPI (blue) to localize the nucleus, TRIC-phalloidin to visualize actin filaments (red), and p-H_2_AX (green) indicative for DNA DSBs^53^.

We next analyzed the expression and phosphorylation of characteristic proteins in the DNA damage response and cell death induction pathways by **Rf**-**1** under light irradiation (Fig. 4a). Capan-1 cells were treated with **Rf**-**1** at a concentration of 10 and 100 µM respectively and 2 mM MES under 1 min of 460-nm irradiation (0.36 J·cm^–2^). Control experiments included the mixture **Rf**-**1** in the dark and cisplatin (100 µM), **Rf** (10 µM), **1** (100 µM) as well as an untreated sample all in the dark and under light irradiation.

As a general remark, light is per se affecting the phosphorylation of the DNA-damage repair-initiating histone H_2_AX. Consequently, H_2_AX phosphorylation was slightly enhanced in all the irradiated samples compared with their corresponding dark analogues, probably suggesting low-level light-mediated DNA damage. Treatment with irradiated **Rf**-**1**, however, led to clear-cut hyperphosphorylation of H_2_AX compared to **Rf**-**1** in the dark, indicating the induction of double strand breaks (DSBs) in DNA. These DSBs in chromatin promptly initiated the phosphorylation of the histone H2A variant to generate p-H_2_AX. This result correlates with the extensive up-regulation observed for cisplatin samples. Nuclear foci in chromatin with accumulated p-H_2_AX can be conveniently detected by immunofluorescence microscopy and serve as beacons of DSB lesions. The respective photomicrographs clearly demonstrated an enhanced number of nuclear p-H_2_AX foci in irradiated cells in presence of **Rf**-**1** indicating efficient cisplatin release (Figs 4b, S23 and S24). Controls of untreated, **Rf**, **1**, **Rf**-**1** and also the photoreaction for **2** and **Rf**-**2** are shown in the supporting information (Fig. S25 and S26). For complex **2**, a similar trend was observed.

Rad51 and rad52 are essential proteins for repair of DNA breaks via homologous recombination^42^ and normally upregulated by DNA damage, e.g. by cisplatin^43^. However, in our experiment both rad51 and rad52 were rather downregulated in Capan-1 cells upon treatment with free cisplatin or **Rf**-**1** under light exposure. The cisplatin-induced downregulation of this repair proteins might be explained by the BRCA2-mutated background of Capan-1 cells, blocking accumulation of rad51/rad52 within DNA damage foci in the cell nucleus^44,45^. This is related to the distinct cisplatin sensitivity of BRCA2-mutant cancer cells^43^. Nevertheless, all these changes demonstrated efficient release of cisplatin upon photoactivation from **Rf**-**1**.

Expression of the DNA damage sensor p53 was already strongly detectable in untreated control cells reflecting the p53 mutated background of Capan-1 cells^46^. Accordingly, p53 levels remained widely stable after the diverse drug exposure regimes. This also explains why the p53 downstream cell cycle regulator p21^47^ was not up- but rather downregulated by DNA-damaging cisplatin and by **Rf**-**1** after light exposure.

Additionally, we investigated the impact of our therapeutic approach on the levels of major vault protein (MVP, also known as lung resistance protein LRP) upregulated by cisplatin and implicated in cisplatin resistance before^48^. MVP levels were generally sensitive to light with reduced expression levels in light-exposed samples. In addition, light exposure abrogated cisplatin-induced MVP upregulation. Interestingly, irradiated **Rf**-**1** samples were, in contrast to the corresponding cisplatin sample, completely devoid of MVP. This suggests that photocatalytic activation strategy for Pt^IV^ prodrugs might help overcoming MVP/LRP-mediated cisplatin resistance^49^.

## Conclusions

The photocatalytic activation of Pt^IV^ complexes by **Rf** is an efficient strategy to control the delivery of active Pt^II^ drugs with low light doses. Cell viability and uptake results obtained in this work evidence for the first time that **Rf** can act as bioorthogonal catalysts towards Pt^IV^ substrates inside the cell. The details of this process within the cellular milieu still require further investigation due to the complexity of **Rf** homeostasis and Pt drugs uptake. Nevertheless, Capan-1 cells, with mutated p53 and a distinct antioxidant cell biology, were effectively treated with our prodrug system **Rf**-**1** based on efficient cisplatin release after only 1 minute of light activation. The induced cell death was clearly mediated by the release and subsequent DNA-damage of cisplatin as proven by light-dependent accumulation of phosphorylated histone H_2_AX in nuclear foci. **Rf**-**1**-mediated cell death specifically suppressed the platinum drug resistance protein MVP/LRP, a facet worth further investigations in the future. This treatment strategy has the potential to be further developed and tested in a photodynamic therapy setting *in vivo*.

## Materials and Methods

### Cell culture

Cell lines SW480 (Dukes’ type B, colorectal adenocarcinoma; ATCC^®^ CCL-228^™^), HCT-116 (colorectal carcinoma; ATCC^®^ CCL-247^™^), A2780 and A2780cis (ovarian carcinoma, Sigma), KB-3-1 (Human papillomavirus-related endocervical adenocarcinoma; HELA derivative, obtained from Dr. D. W. Shen, Bethesda, Maryland) VM47 (primary melanoma as described previously^50^), H35 and H52 (primary glioblastoma as described previously^51^) and Capan-1 (liver metastatic pancreatic cancer; ATCC^®^ HTB-79^™^) were grown as a monolayer at 37 °C in a humified 5% CO_2_ atmosphere. RPMI growth medium was supplemented with 10% fetal bovine serum (PAA, Linz, Austria). Cell cultures were periodically checked for Mycoplasma contamination. All media were supplemented with 2 mM MES buffer (pH 6, 1% vol) or H_2_O (1% vol).

### MTT assay of the cell viability

In a typical experiment, 4 × 10^4^ cells/ml (5 × 10^4^ cells/ml in the case of Capan-1 only) were seeded in 96-well plates in 100 µL of cell culture medium. After 48 h, cells were administered with the anticancer agents at the chosen concentrations, under dark conditions. After 1 h, cells were irradiated during 1 min at 460 nm (6 mW·cm^–2^, corresponding to a light dose of 0.36 J·cm^–2^) and left in the incubator for 6 h. Dark analogues were directly incubated 7 h. Cell culture media was replaced by fresh media and incubated for a total of 72 h. After this incubation period, cell viability was determined by means of the MTT assay following the instructions of the manufacturer (EZ4U Cell Proliferation and Cytotoxicity assay, from Biomedica Medizinprodukte GmbH & Co KG).

### Pt uptake in Capan-1 cells measured by ICP-MS

Capan-1 cells in 6 well-plate with a concentration of 3 × 10^5^ cells/well were seeded and after 48 h exposed to cisplatin, **1** and **Rf**-**1** under different incubation conditions. In different set of experiments, cells were exposed to Pt agents as follows: a) 1 h in the dark, b) 1 h preincubation in the dark +1 min of 460-nm light irradiation +3 h in the dark, c) 1 h preincubation in the dark +1 min of 460-nm light irradiation +6 h in the dark, d) 1 min of 460-nm light irradiation +7 h in the dark. All dark controls were not light irradiated and protected from ambient light. The digestion protocol adopted was described by Keppler and coworkers previously^52^.

### Circular Dichorism

Circular dichroism spectra were recorded on Chirascan™ CD (by Applied Photophysics) equipped with a single cell Peltier temperature controller. All the measurements were performed using 1 cm path-length quartz cuvettes and setting the following parameters: range 200–500 nm; step 1 nm; time per point 0.1 s; repeats 4; temperature 25 °C. Concentration of *ds26*, **Rf** and **1** were adjusted so that the HV (HT) voltage could be properly controlled, giving reliable ellipticity values over the investigated wavelength range (HV always ≤ 550, being 1000 the maximum suitable value for the Chirascan CD instrument). The lyophilized *ds26* was firstly diluted in IDTE buffer (10 mM Tris, pH 7.5, 0.1 mM EDTA, Integrated DNA Technologies) to obtain 100 μM stock solution. The annealing process to ensure the 100% formation of the B-DNA conformation was performed by heating the *ds26* stock solution to 95 °C for 5 min, followed by slowly cooling to room temperature overnight. Stock solution of *ds26*, together with stock solutions of cisplatin, **Rf**, **1**, succinic acid, were used to prepare the final mixtures at the required concentrations diluting them with MES buffer. After irradiation for 5 minutes, the final solutions have been incubated at 37 °C for 24 or 48 h and then measured.

The oligonucleotide *ds26* 5′-CAATCGGATCGAATTCGATCCGATTG-3′ (GC content 46.2%) was purchased from IDT (Integrated DNA Technologies) in HPLC purity grade.

### Pt-NMR

NMR measurements were recorded on a Brucker Avance III 500 MHz spectrometer at 500.32 (^1^H) and 107.38 (^195^Pt) MHz at 25 °C. ^195^Pt resonances were referenced relative to external K_2_[PtCl_4_].

### Mass spectrometry

Mass spectrometry detection was carried out using a time-of-flight mass spectrometer (ESI-TOF) LCT Premier XE from Waters (Milford, MA, USA) with an electrospray ionization source, working in positive/W centroid mode. The m/z acquisition range was between m/z 100–1000. The capillary and cone voltages were set at 2000 and 50 V, respectively. For other parameters, desolvation gas temperature was 200 °C and source temperature was 100 °C. The desolvation gas flow was set at 600 Lh^−1^ and cone gas flow was set at 30 Lh^−1^. Samples were prepared in water and were infused directly into the mass spectrometer at 10 μLmin^−1^. Masslynx v4.1 software was used to analyze the spectra (Waters, Milford, MA, USA).

MALDI-TOF-MS analysis was performed using an UltrafleXtreme III time-of-flight mass spectrometer equipped with a Nd:YAG laser (Smartbeam II, 355 nm, 1 kHz) and controlled by Flex Control 3.3 software (Bruker Daltonics, Bremen, Germany). The acquisitions were carried out in positive-ion linear mode at a laser frequency of 1 kHz. The spectrum was acquired at 50% laser fluency and was recorded in the m/z range from 5000 to 10000. The deflector cutoff was set at m/z 4500 and the spectrum resulted from accumulation of 1500 laser shots. All the spectra were analyzed using FlexAnalysis software 3.0 (Bruker Daltonics, Bremen, Germany). Sample preparation was carried out by deposition of 0.5 µL of the final mix directly onto a polished stainless-steel plate (Bruker Daltonics, Bremen, Germany) and 0.5 µL of matrix solution. The matrix solution was prepared after dissolving 50 mg of 3-hydroxypicolinic and 5 mg of diammonium citrate in deionized water. The oligonucleotide ssODN 5′-AAAGAGAACGCTAAGAGGAGGAA-3′ was purchased from IDT (Integrated DNA Technologies) in PAGE purity grade.

### Western blot analysis

Preparation of samples: total cell protein extracts were separated by 10% sodium dodecyl sulfate- polyacrylamide gel electrophoresis (SDS-PAGE) and blotted onto polyvinylidene difluoride membranes (PVDF, Thermo Fisher Scientific). Anti-ß-actin antibody (AC-15) was purchased from Sigma, anti-rad51 (#8875), anti-rad52 (#3425), anti-p21 (#2947) and anti-p-H_2_AX (#9718) from Cell Signaling Technology, and anti-p53 (DO-1) from Thermo Fisher Scientific and anti-MVP (#ALX-801–005) from Enzo Life Science. Secondary anti-mouse (#7076) and anti-rabbit (#7074) horseradish peroxidase-labeled antibodies were obtained from Cell Signaling Technologies.

### Immunofluorescence Microscopy

3 × 10^4^ cells/well were seeded in 8-well spot slides (Thermo Fisher Scientific). After 48 h, cells were co-incubated for 1 h with the different concentrations of cisplatin, **1**, **Rf**, **Rf**-**1** in darkness, in duplicates. Then, one slide was kept in the dark for 6 hours more and the other was irradiated during 1 minute at 460 nm with an LED light source (6 mW·cm^–2^), and also kept in the dark for 6 hours. Cells were rinsed with PBS and fixed with MeOH/Acetone 1:1 during 20 min at −20 °C, rinsed again with PBS and blocked with PBS + 10% BSA for 1 h. Blocking solution was aspirated and the diluted primary p-H_2_AX Antibody 1:200 was applied and incubated at 4 °C overnight. After rinsing with PBS, secondary antibody goat anti-rabbit antibody conjugated to AlexaFluor488 (1:500) (Thermo Fisher) was co-incubated with phalloidin (1:500) and DAPI (1.5 µg/mL) for 1 h. Images were acquired on an inverted point scanning confocal microscope with PMTs (LSM700, Zeiss, Jena, Germany) using a 63×Plan-Apochromat 63×/1.4 oil immersion objective with Zen2010® software (Zeiss) using 405 nm (DAPI), 488 nm (AlexaFluor488) or 555 nm (phalloidin) solid state laser lines for excitation and 555 nm short pass (for DAPI and AlexaFluor488) and 560 nm long pass (phalloidin) emission filters, respectively.

## Electronic supplementary material

Supplementary Information
