# Supplementary material for: Biological activity of PtIV prodrugs triggered by riboflavin-mediated bioorthogonal photocatalysis
Source: Sci Rep. 2018 Nov 21;8:17198. doi: 10.1038/s41598-018-35655-2 (PMC6249213; doi:10.1038/s41598-018-35655-2)
Supplement: Supplementary file 1 — Supplementary Information [file 41598_2018_35655_MOESM1_ESM.pdf]

Supporting Information

Biological activity of Pt^{IV} prodrugs triggered by riboflavin-mediated bioorthogonal photocatalysis

Silvia Alonso-de Castro,[a] Alessio Terenzi,[b,c,d] Sonja Hager,[e] Bernhard Englinger,[e] Adriana Faraone,[b] Javier Calvo Martínez,[a] Mathea Sophia Galanski,[c] Bernhard K. Keppler,[c] Walter Berger*[e] & Luca Salassa*[b,f]

[a] *CIC biomaGUNE, Paseo de Miramón 182, Donostia, 20014 (Spain)*
[b] *Donostia International Physics Center, Paseo Manuel de Lardizabal 4, Donostia, 20018 (Spain)*
[c] *Institute of Inorganic Chemistry, University of Vienna, Waehringerstrasse 42, A-1090 Vienna (Austria)*
[d] *Research Platform "Translational Cancer Therapy Research", University of Vienna and Medical University of Vienna, Vienna (Austria)*
[e] *Institute of Cancer Research and Comprehensive Cancer Center, Medical University of Vienna, Borschkegasse 8a, Vienna (Austria)*
[f] *Ikerbasque, Basque Foundation for Science, Bilbao, 48011 (Spain)*

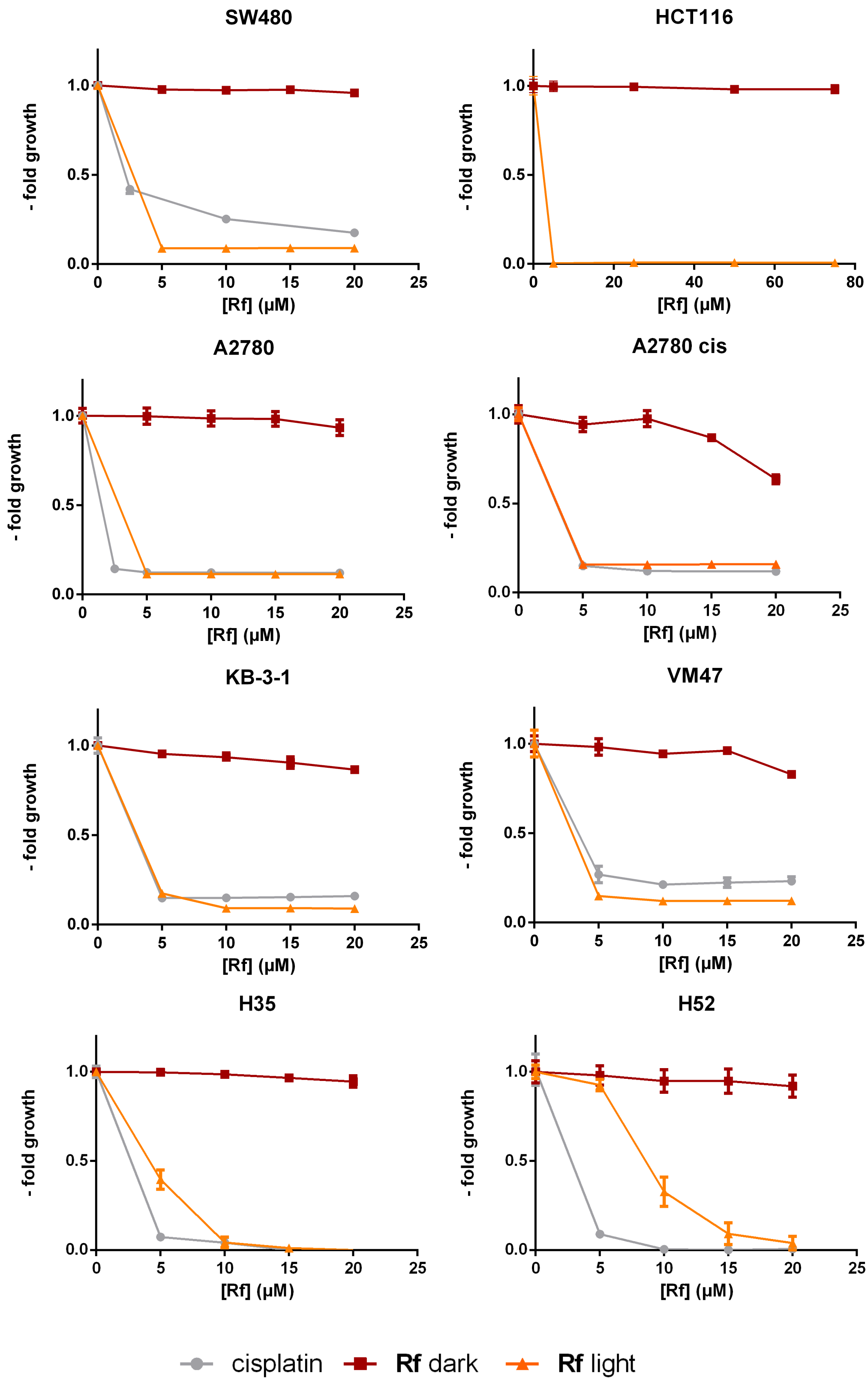

Figure S1. Phototoxic effect of **Rf** (2 mM MES) on the cell viability of different cell lines after 72 h with and without light irradiation (460 nm, 0.36 J·cm^{-2}). Cisplatin was employed as positive control (10x [**Rf**]).

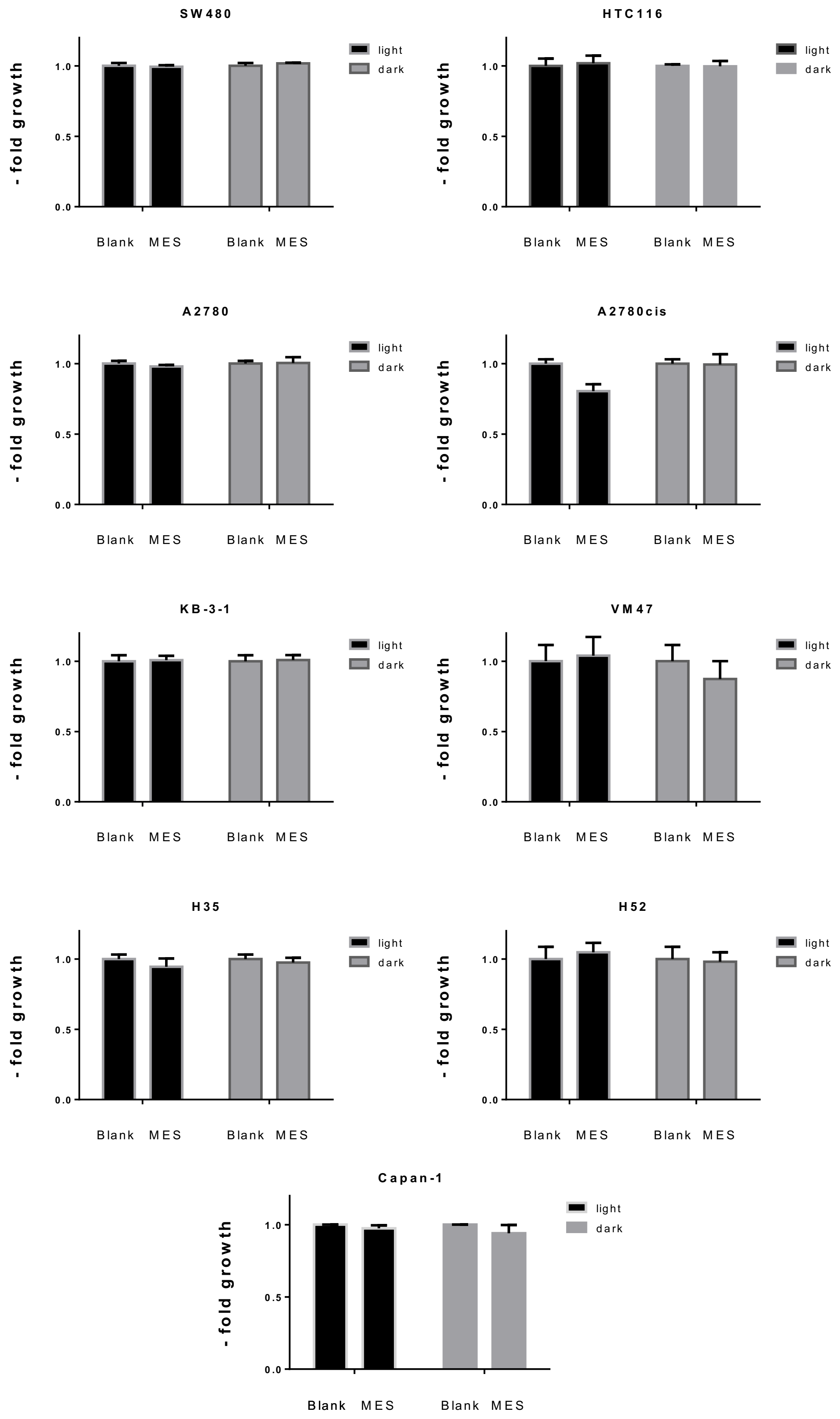

Figure S2. Cell viability of different cell lines in the presence of 2 mM MES after 72 h with and without light irradiation (460 nm, 0.36 J·cm^{-2}).

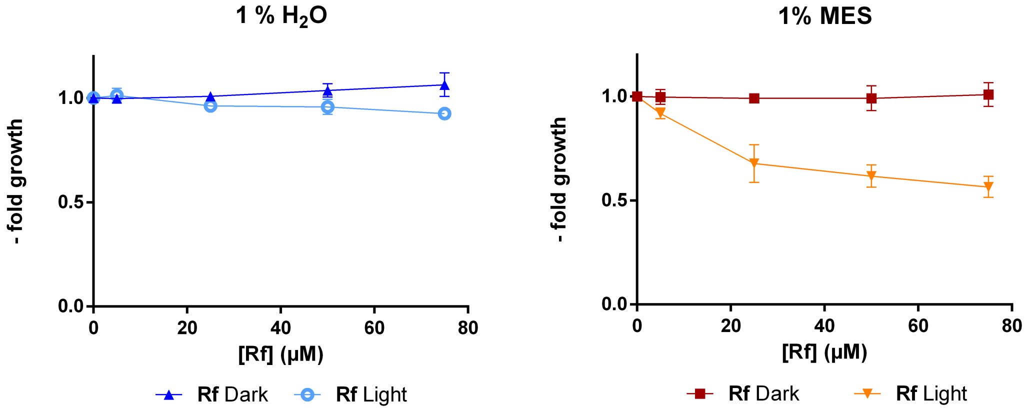

Figure S3. **Rf** phototoxic effect on the cell viability of Capan-1 after 72 h in the absence (1% H_2O) and in the presence of 2 mM MES (1%). Experiments were performed in the dark and under light irradiation (460 nm, 0.36 J·cm^{-2}).

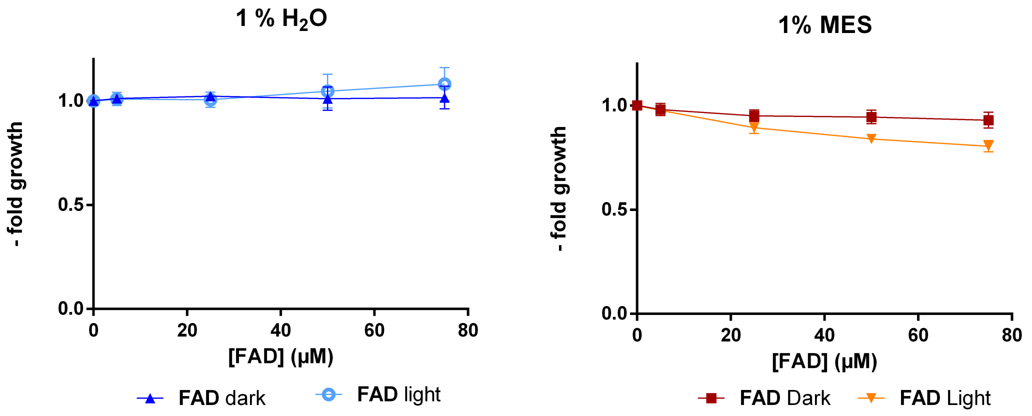

Figure S4. **FAD** phototoxic effect on the cell viability of Capan-1 after 72 h in the absence (1% H_2O) and in the presence of 2 mM MES (1%). Experiments were performed in the dark and under light irradiation (460 nm, 0.36 J·cm^{-2}).

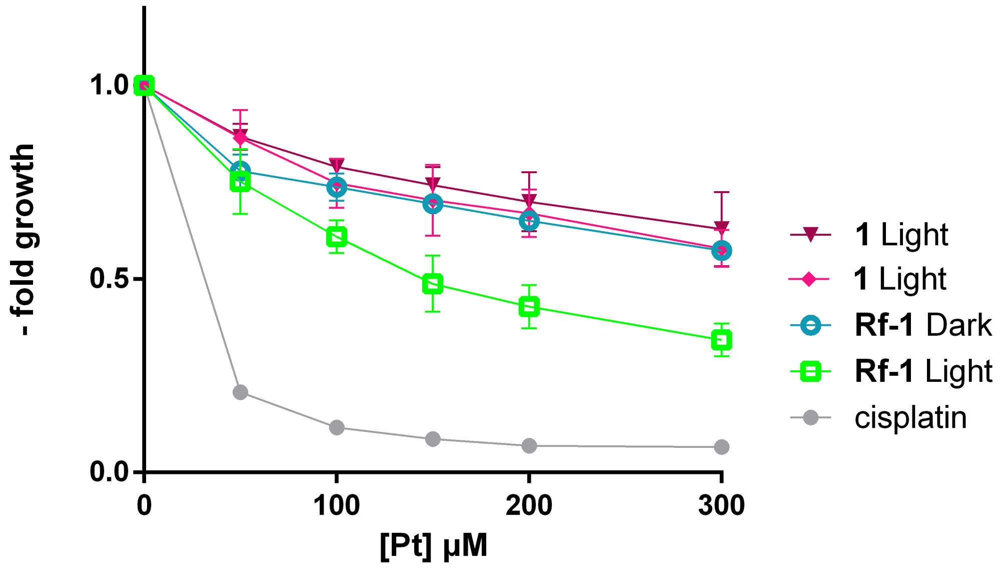

Figure S5. Photocatalytic **Rf-1** effect against Capan-1 cells. Cell viability of Capan-1 upon incubation with **Rf-1** (1:10) and **1** in the absence of MES under light irradiation (460 nm, 0.36 J·cm^{-2}) and in the dark. Cisplatin is shown as positive control.

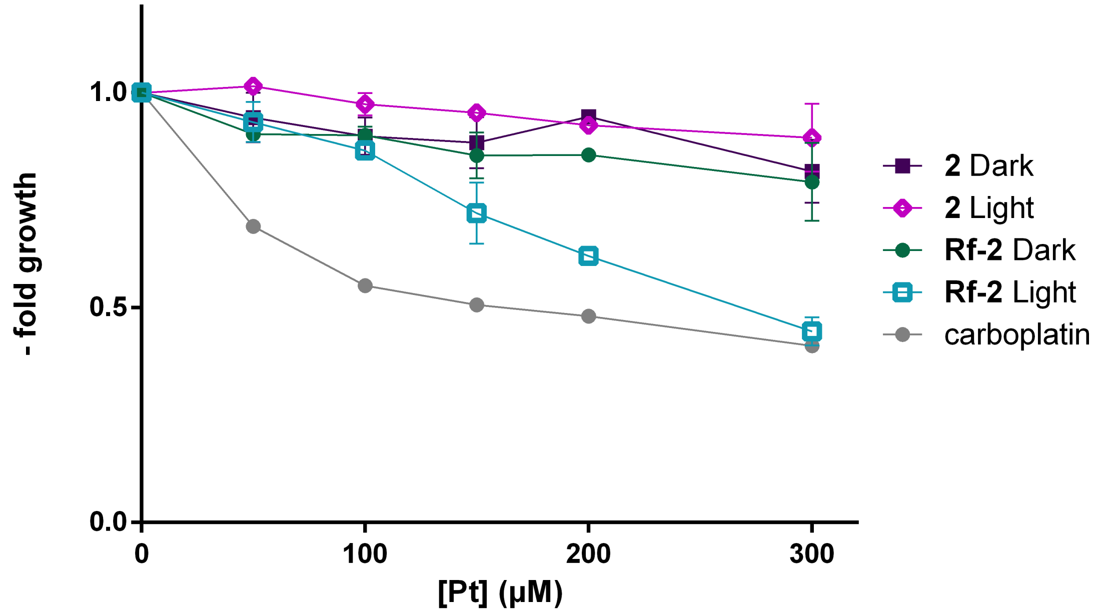

Figure S6. Photocatalytic **Rf-2** effect against Capan-1 cells. Cell viability of Capan-1 upon incubation with **Rf-2** (1:10) and **2** in the absence of MES under light irradiation (460 nm, 0.36 J·cm^{-2}) and in the dark (n = 2). Carboplatin is shown as positive control.

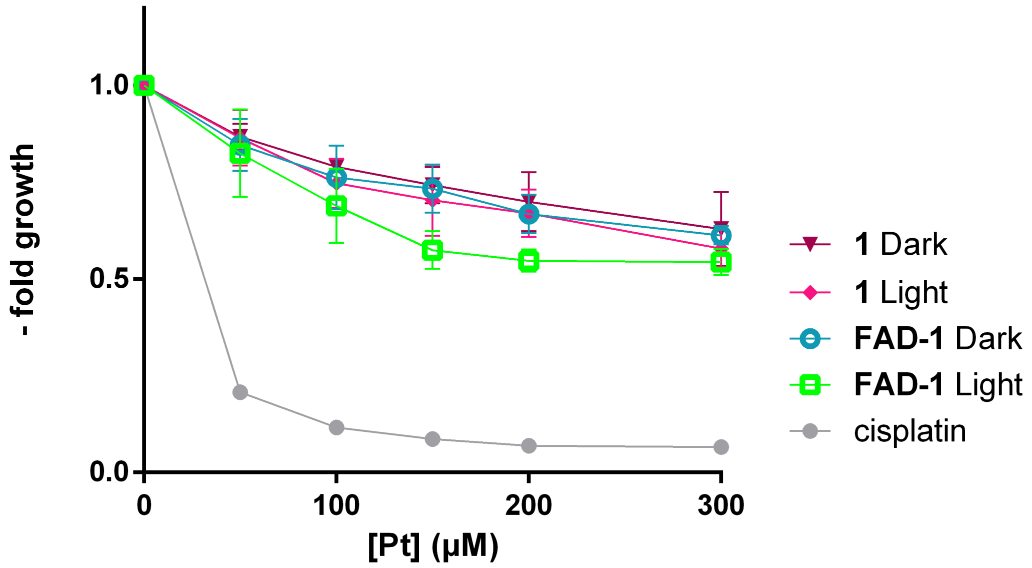

Figure S7. Photocatalytic **FAD-1** effect against Capan-1 cells. Cell viability of Capan-1 upon incubation with **FAD-1** (1:10) and **1** in the absence of MES under light irradiation (460 nm, 0.36 J·cm^{-2}) and in the dark. Cisplatin is shown as positive control.

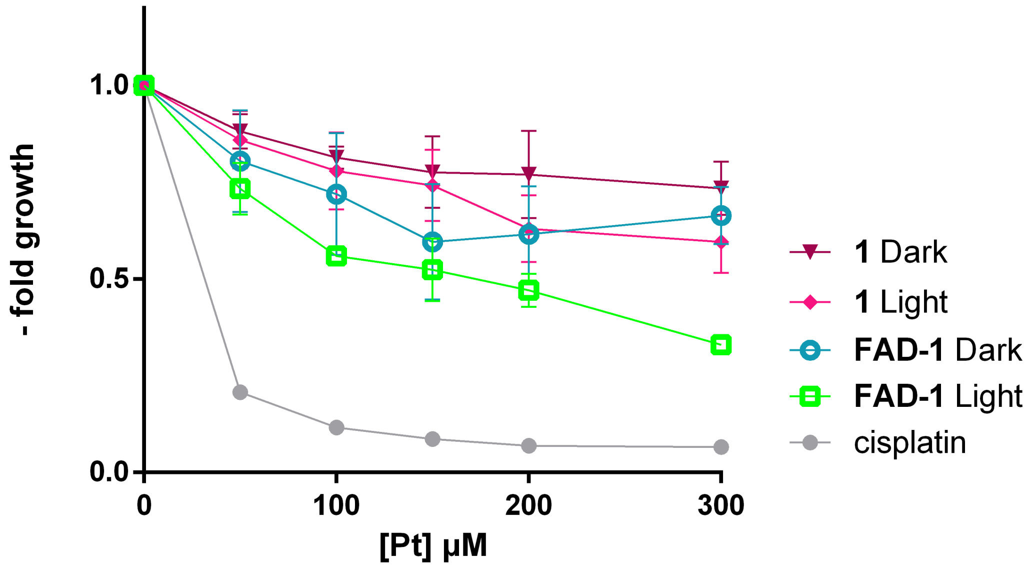

Figure S8. Photocatalytic **FAD-1** effect against Capan-1 cells. Cell viability of Capan-1 upon incubation with **FAD-1** (1:10) and **1** in the presence of 2 mM MES under light irradiation (460 nm, 0.36 J·cm^{-2}) and in the dark. Cisplatin is shown as positive control.

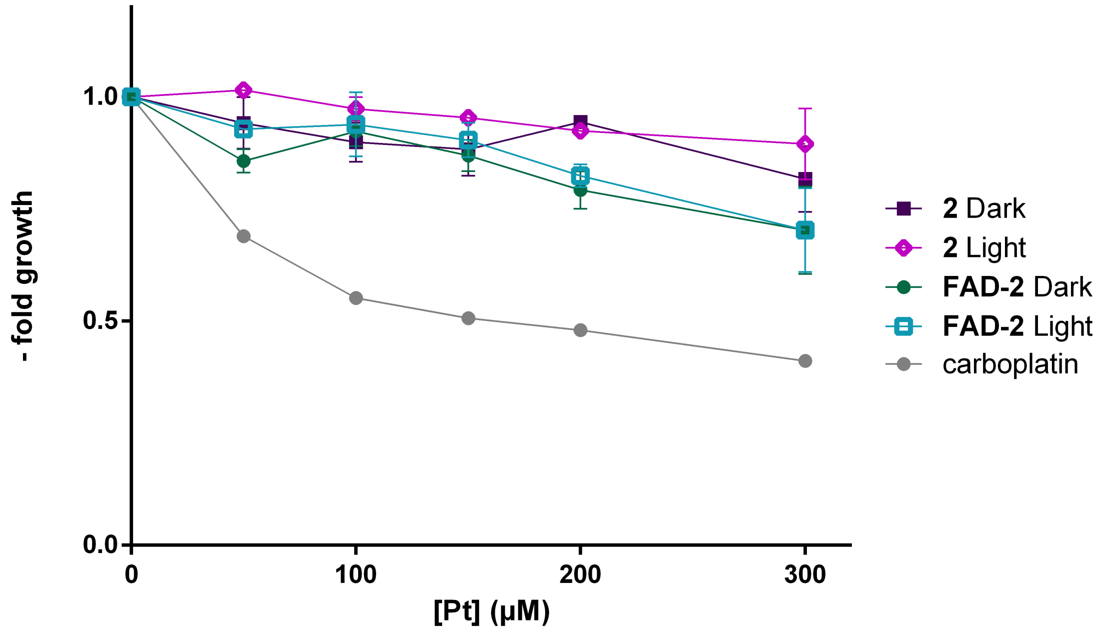

Figure S9. Photocatalytic **FAD-2** effect against Capan-1 cells. Cell viability of Capan-1 upon incubation with **FAD-2** (1:10) and **2** in the absence of MES under light irradiation (460 nm, 0.36 J·cm^{-2}) and in the dark. Carboplatin is shown as positive control.

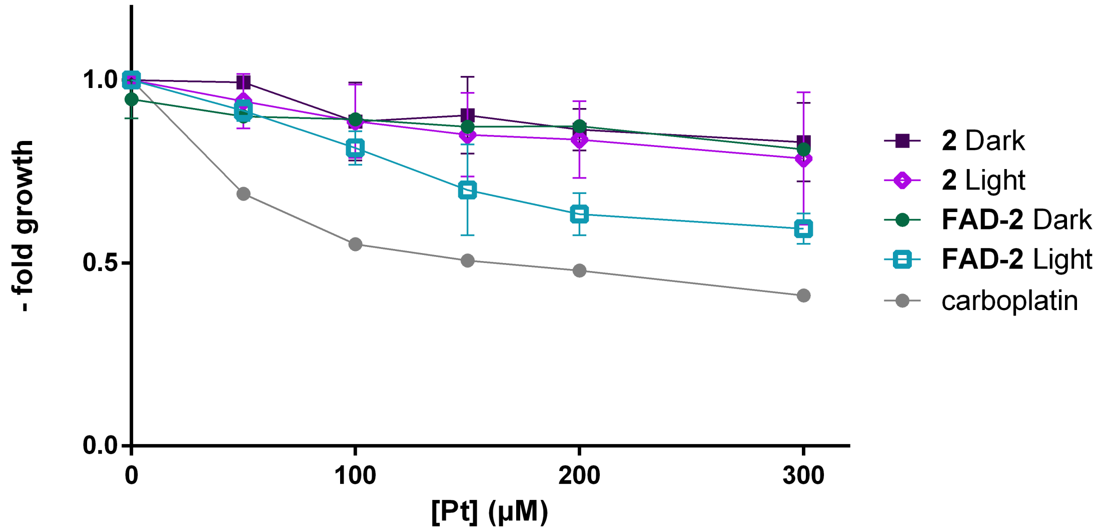

Figure S10. Photocatalytic **FAD-2** effect against Capan-1 cells. Cell viability of Capan-1 upon incubation with **FAD-2** (1:10) and **2** in the presence of 2 mM MES under light irradiation (460 nm, 0.36 J·cm^{-2}) and in the dark. Carboplatin is shown as positive control.

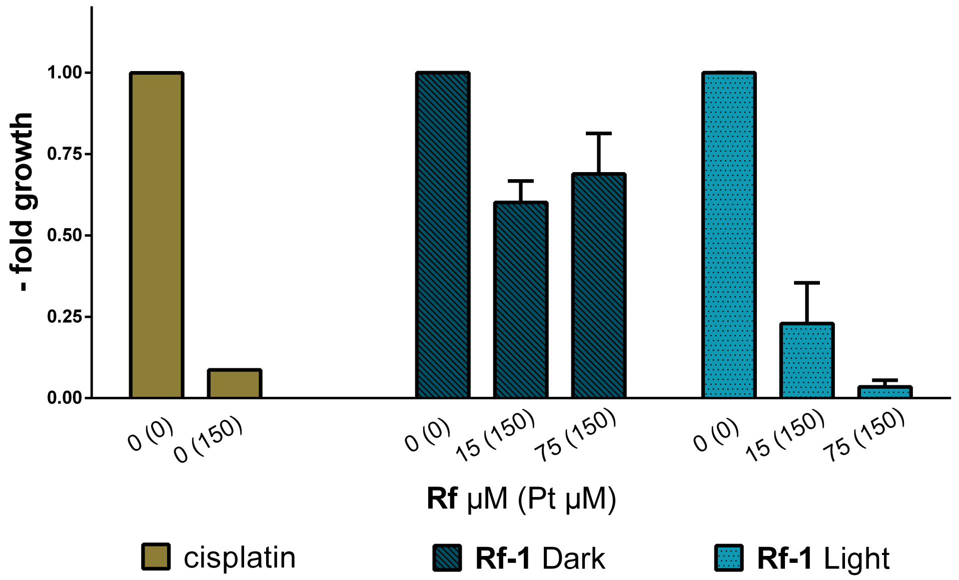

Figure S11. Cell viability of Capan-1 cells treated with two different **Rf-1** ratios (1:10 and 1:2) with 2 mM MES, under light irradiation (460 nm, 0.36 J·cm^{-2}) and in the dark. Cisplatin is shown as positive control.

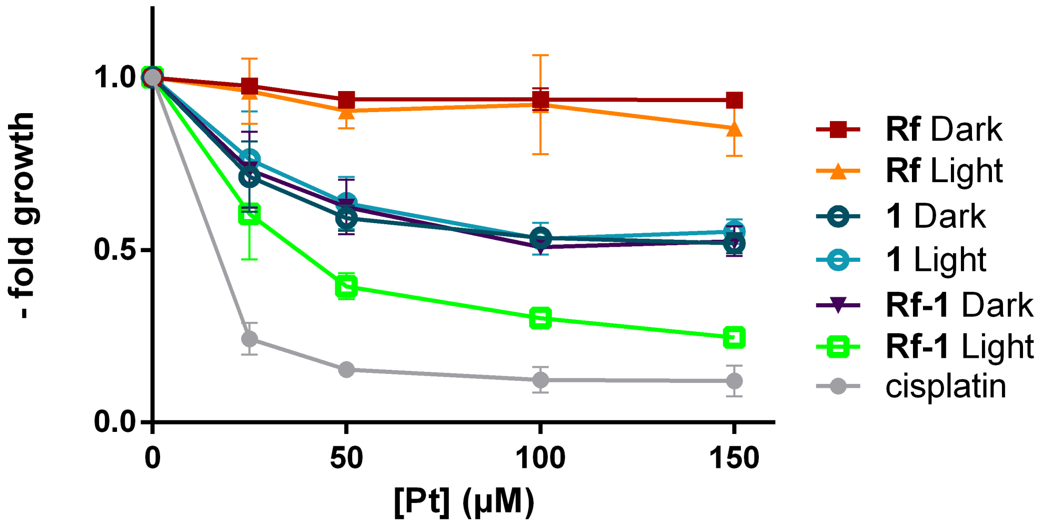

Figure S12. Photocatalytic **Rf-1** effect against Capan-1 cells in hypoxia conditions. Cell viability of Capan-1 cells upon incubation with **Rf-1** (1:10), **Rf** and **1** in the presence of 2 mM MES, under light irradiation (460 nm, 0.36 J·cm^{-2}1 min at 6 mW cm^{-2}) and in the dark. Cisplatin is shown as positive control.

**Structure**	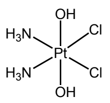	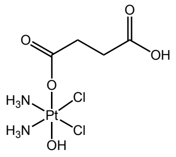	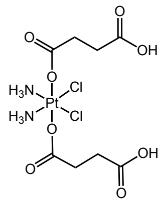	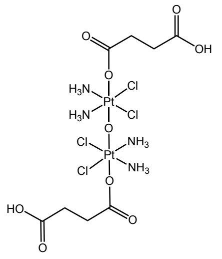
**Theoretical Isotopic Pattern**	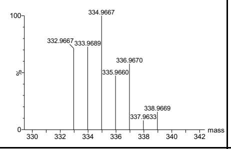	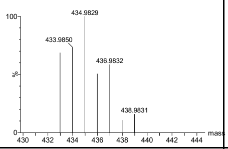	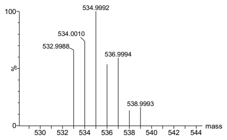	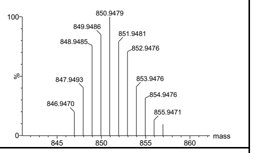
**Experimental Isotopic Pattern**	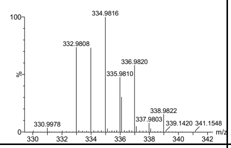	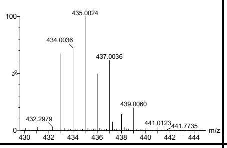	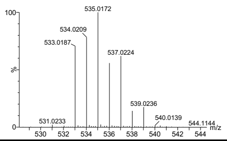	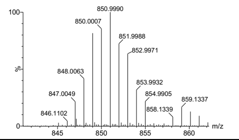
**m/z**	335	435	535	851
	$[M+H]^+$	$[M+H]^+$	$[M+H]^+$	$[M+H]^+$
	**Peak Intensity**	**Peak Intensity**	**Peak Intensity**	**Peak Intensity**
t_{irrad}=0 min	1.53e3	1.63e2	1.51e5	6.44e2
t_{irrad}=2 min	1.24e4	2.02e4	1.30e5	9.39e3
t_{irrad}=5 min	1.99e4	2.79e4	1.07e5	1.55e4
t_{irrad}=10 min	6.27e4	5.77e4	4.11e4	2.02e4

Figure S13. Selected mass spectrometry peak and their relative intensity obtained by irradiating at 460 nm (0, 2, 5 and 10 min) a solution of 2.7 mM **1** and 267 µM **Rf** (ratio **Rf**:**1** 1:10) dissolved in 2 mM MES buffer (pH 6).

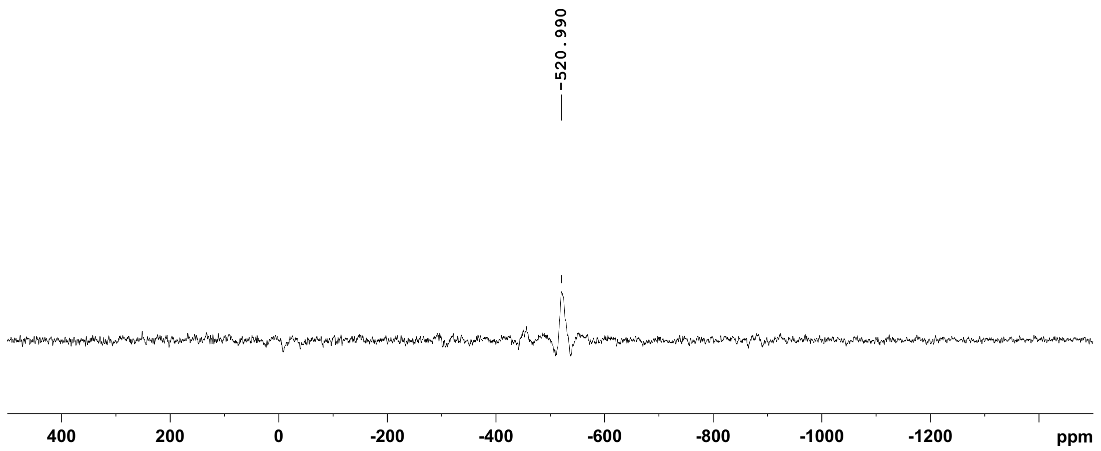

Figure S14. ^{195}Pt-NMR spectrum of cisplatin. A solution of 2.7 mM cisplatin dissolved in 2 mM MES buffer (pH 6) was measured.

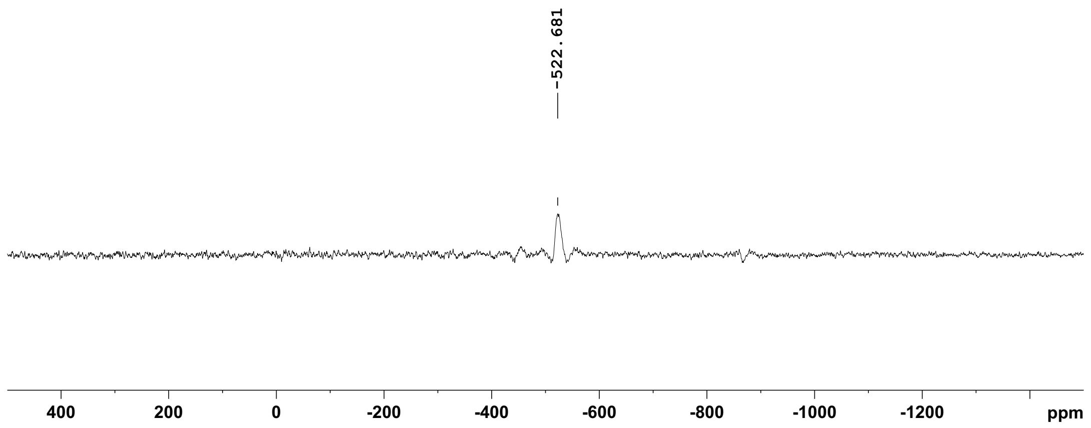

Figure S15. ^{195}Pt-NMR spectrum of cisplatin. A solution of 2.7 mM cisplatin and 267 µM **Rf** (ratio **Rf**:cisplatin 1:10) dissolved in 2 mM MES buffer (pH 6) was measured.

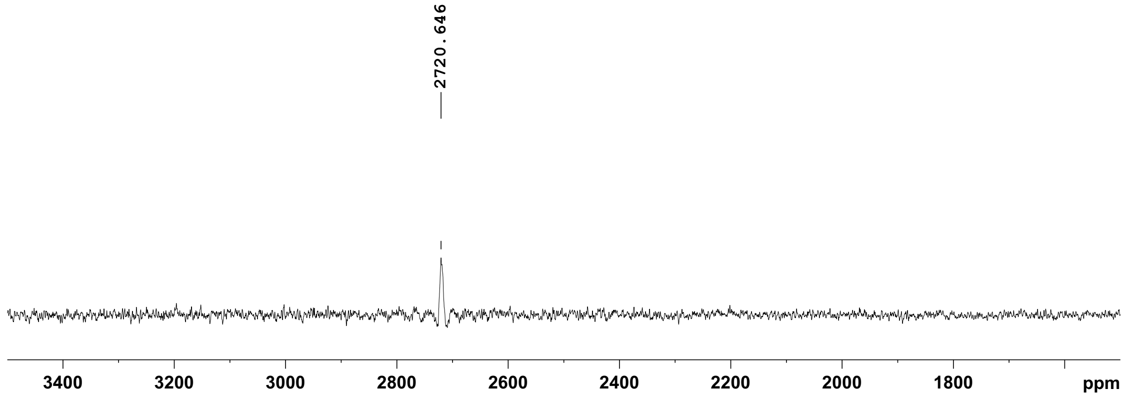

Figure S16. ^{195}Pt-NMR spectrum. A solution of 7.2 mM **1** and 267 µM **Rf** (ratio **Rf**:**1** 1:26.7) were dissolved in 2 mM MES buffer (pH 6) was measured in the dark.

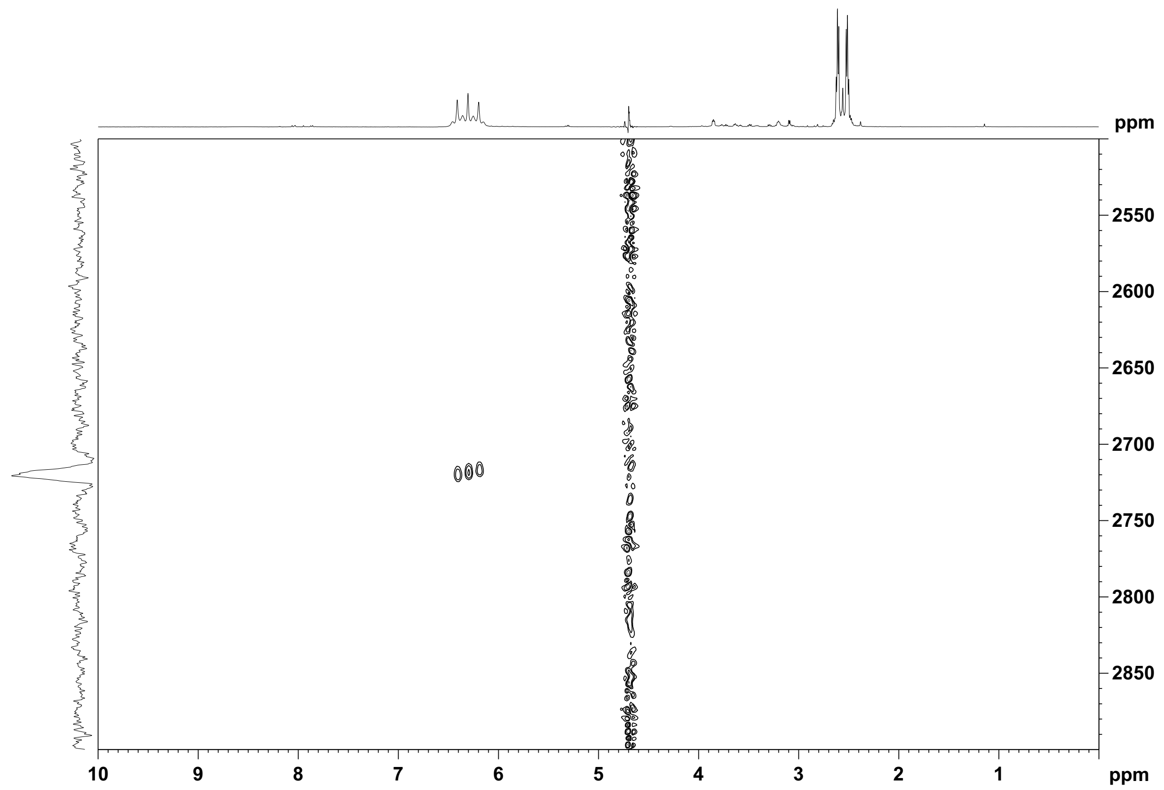

Figure S17. ^{1}H,^{195}Pt-HSQC NMR spectrum. A solution of 7.2 mM **1** and 267 µM **Rf** (ratio **Rf**:**1** 1:27) dissolved in 2 mM MES buffer (pH 6) was measured in the dark.

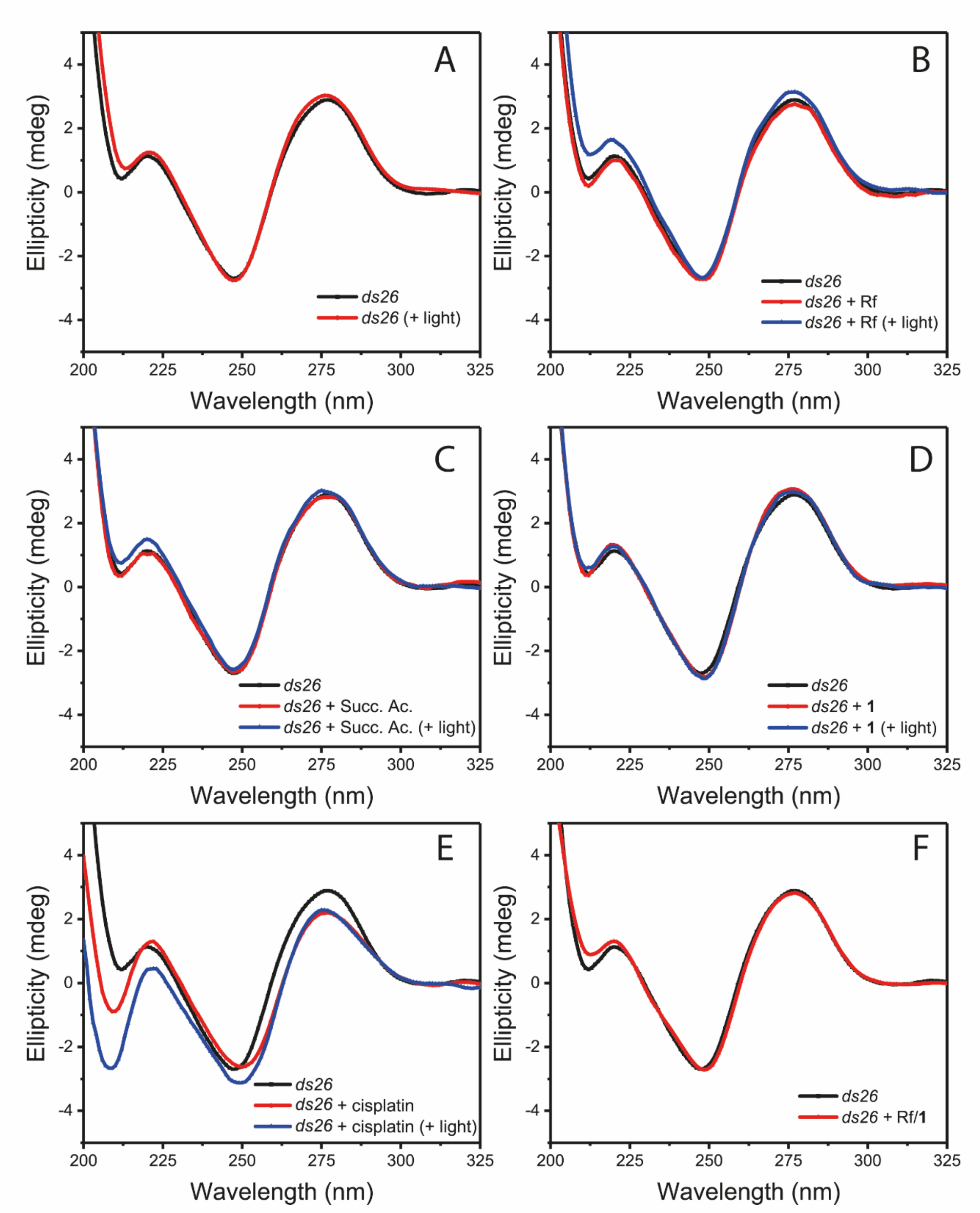

Figure S18. CD spectra of a) 1 µM *ds26* in the dark and after irradiation; b) 1 µM *ds26* in combination with 0.3 µM **Rf** in the dark and after irradiation; c) 1 µM *ds26* in combination with 6 µM succinic acid in the dark and after irradiation; d) 1 µM *ds26* in combination with 3 µM **1** in the dark and after irradiation; e) 1 µM *ds26* in combination with 3 µM cisplatin in the dark and after irradiation; f) 1 µM *ds26* in combination with 3 µM **1** and 0.3 µM **Rf** in the dark. Incubation: 48h at 37 °C. Buffer: MES 20 mM (pH=6.0). Light irradiations were performed at 460 nm (0.36 J·cm^{-2}).

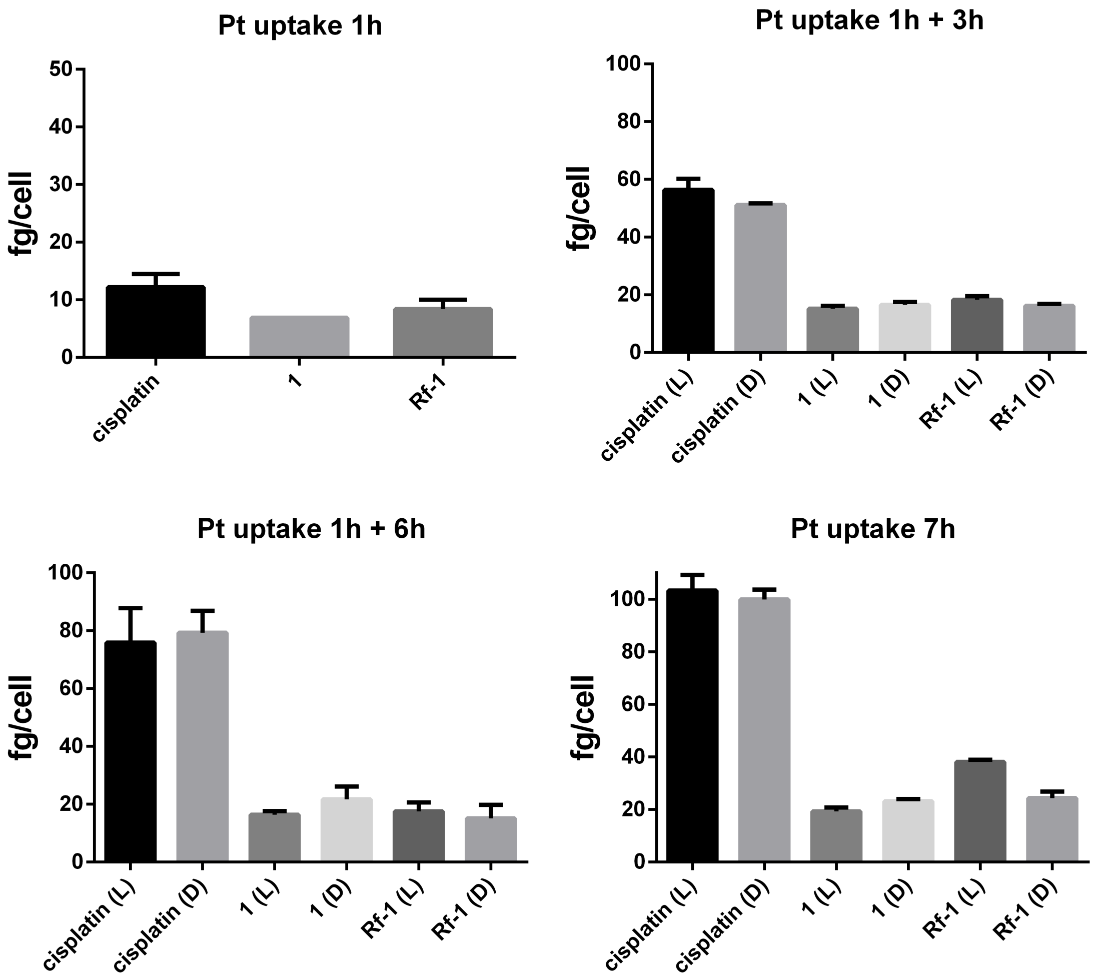

Figure S19. Pt accumulation in Capan-1 cells measured by ICP-MS at different time points. Cells treated with 100 µM cisplatin, 100 µM **1** and 10:100 µM **Rf-1** were incubated a) 1 h in the dark, b) 1 h preincubation in the dark + 1 min of 460-nm light irradiation + 3 h in the dark, c) 1 h preincubation in the dark + 1 min of 460-nm light irradiation + 6 h in the dark, d) 1 min of 460-nm light irradiation + 7 h in the dark. All dark controls were not light irradiated and protected by ambient light.

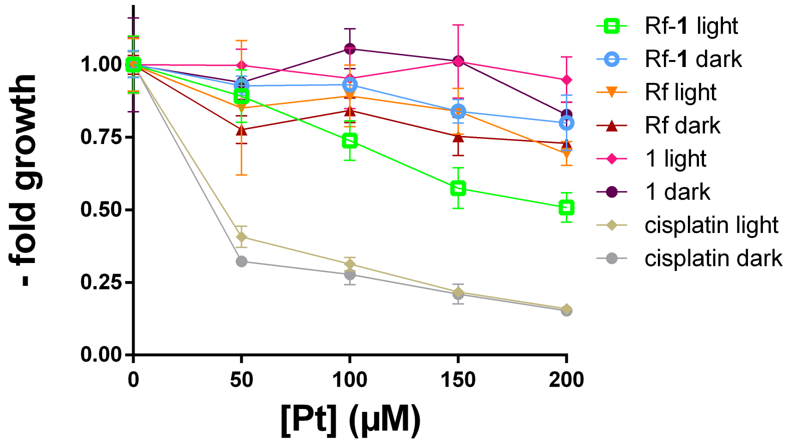

Figure S20. Photocatalytic **Rf-1** effect against Capan-1 cells. Cell viability of Capan-1 cells upon incubation with **Rf-1** (1:10), **Rf** and **1** in the presence of 2 mM MES during 1 h and followed by light irradiation (460 nm, 0.36 J·cm^{-2}). Media was renewed immediately after irradiation. Dark controls were performed accordingly. Cisplatin is shown as positive control.

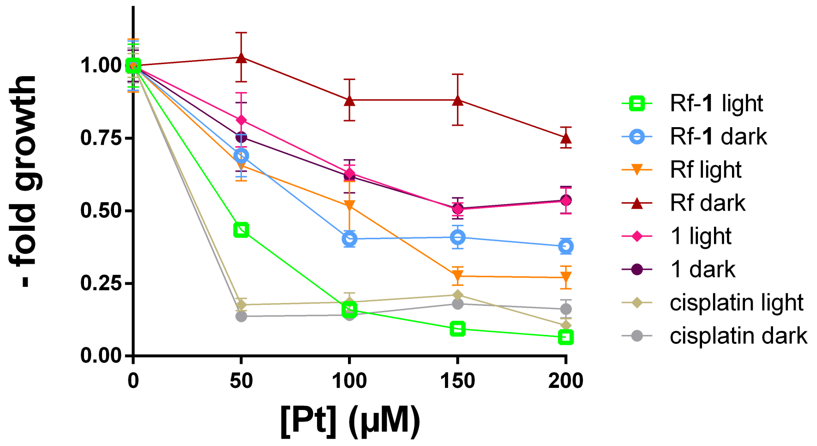

Figure S21. Photocatalytic **Rf-1** effect against Capan-1 cells. Cell viability of Capan-1 cells upon incubation with **Rf-1** (1:10), **Rf** and **1** in the presence of 2 mM MES were irradiated (460 nm, 0.36 J·cm^{-2}) and incubated for 7 h. Media was renewed after these 7 h. Dark controls were performed accordingly. Cisplatin is shown as positive control.

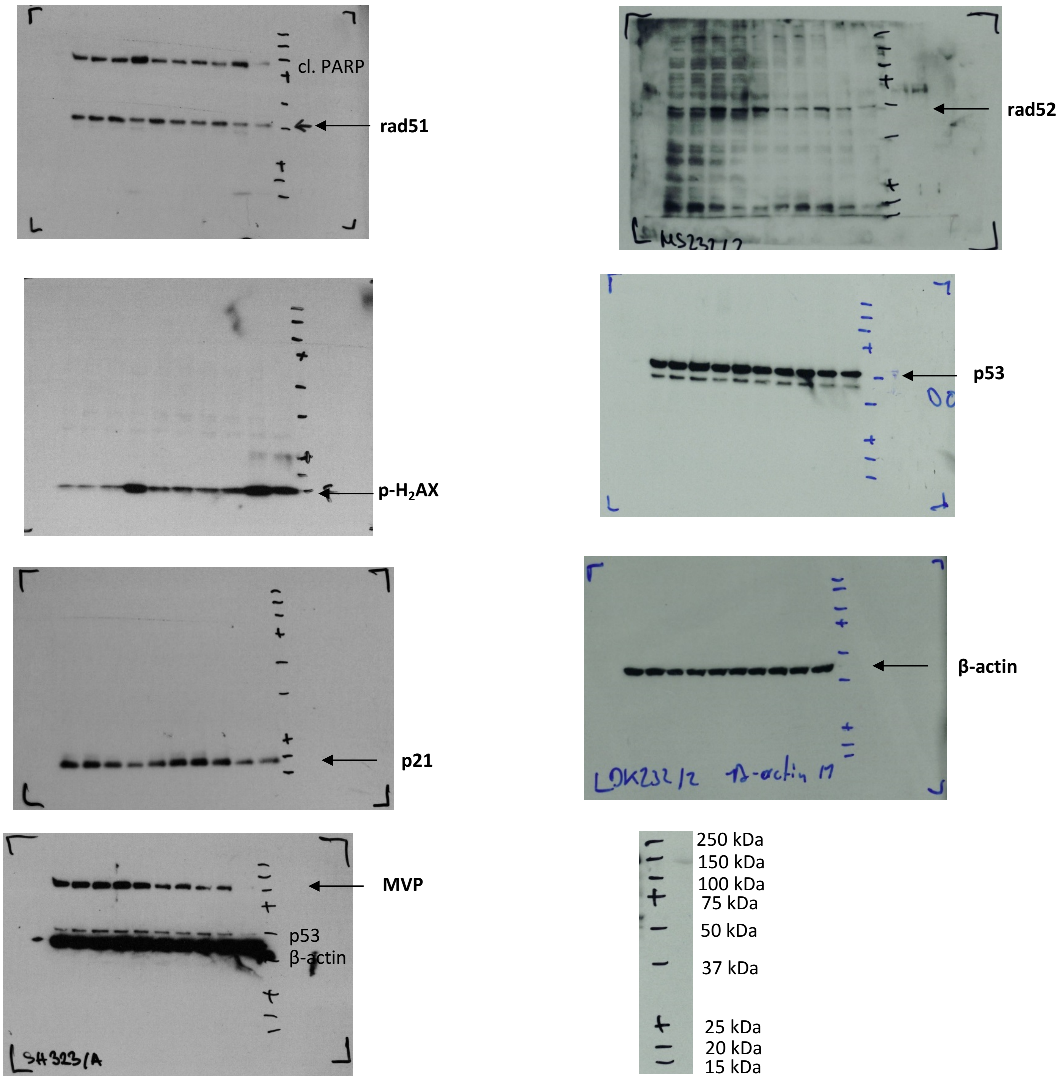

Figure S22. Original films for protein detection by Western blot analyses. Bands shown in Figure 4a of the main manuscript are indicated in bold face and by arrows.

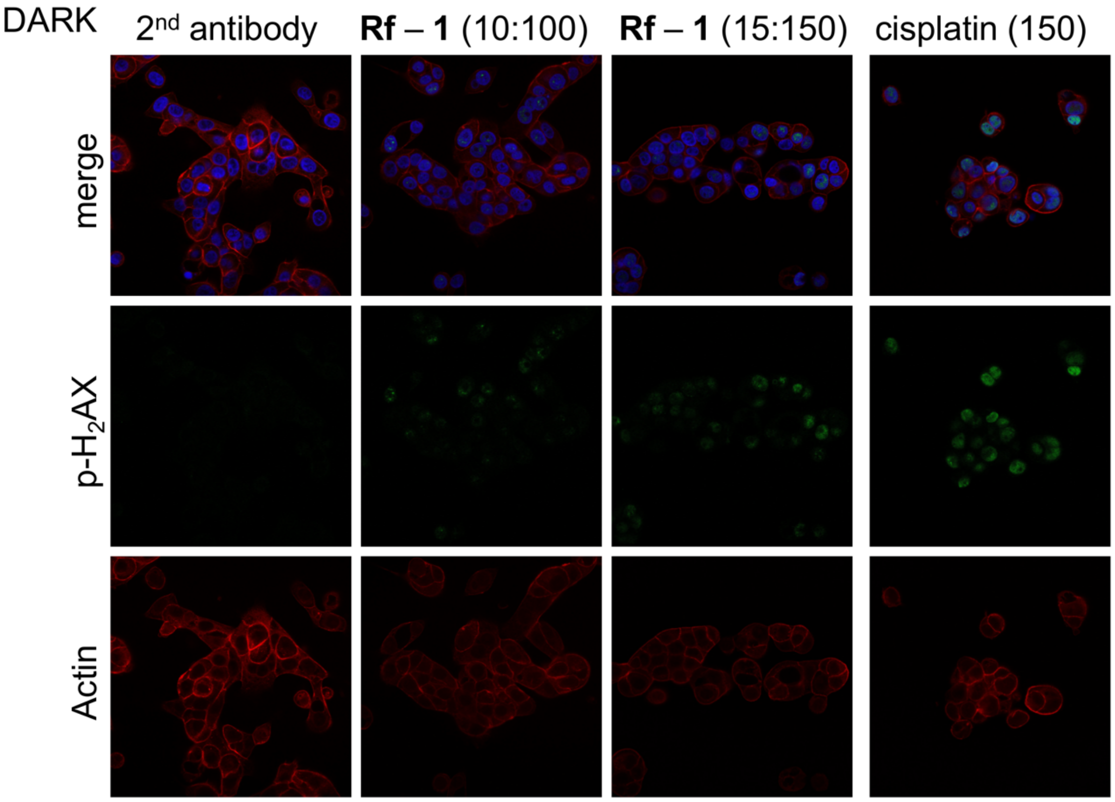

Figure S23. Immunofluorescence microscopy images of Capan-1 cells. Images correspond to control samples: **Rf** (15 µM), **Rf-1** (10–100 µM and 15–150 µM) and cisplatin (150 µM) under dark conditions. Cells were stained using DAPI for nuclei localization (blue channel), TRIC-phalloidin to visualize actin filaments (red channel) and primary antibody histone H_2AX antibody (green channel).

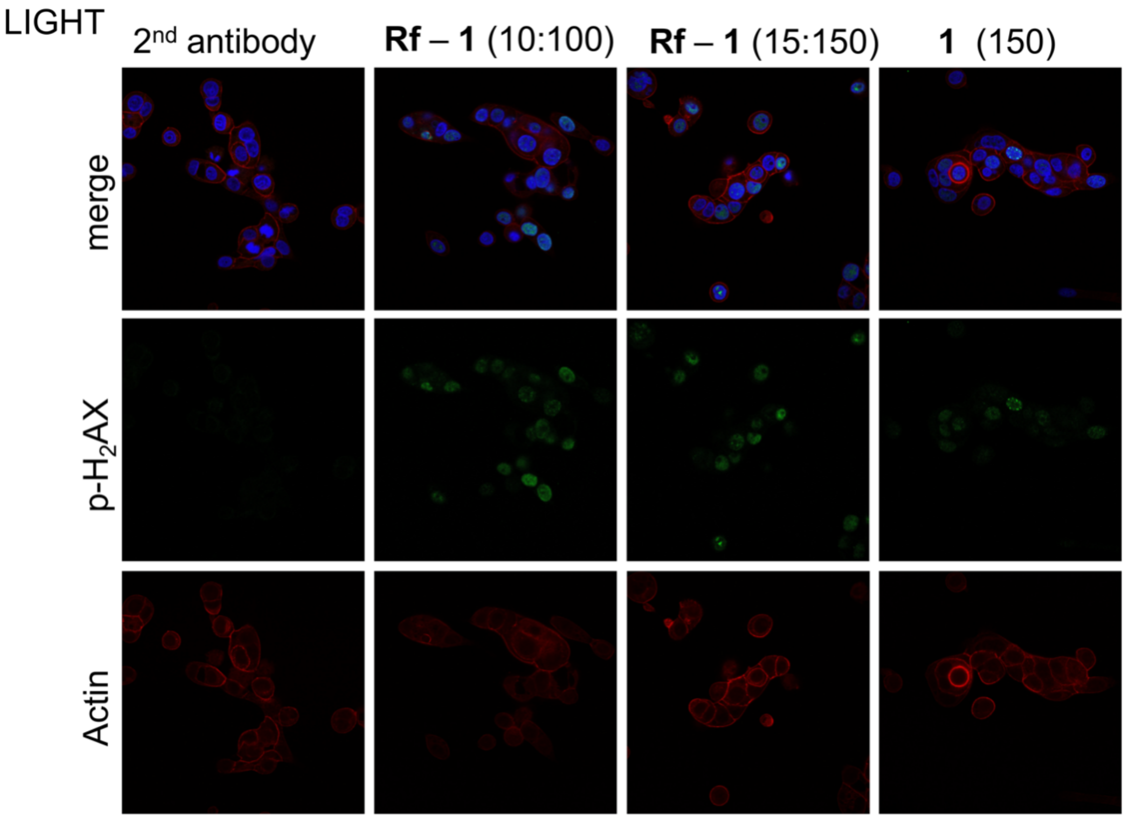

Figure S24. Immunofluorescence microscopy images of Capan-1 cells. Images correspond to control sample: **Rf-1** (10–100 µM and 15–150 µM) and **1** (150 µM) irradiated 1 min at 460 nm (0.36 J·cm^{-2}). Cells were stained using DAPI for nuclei localization (blue channel), TRIC-phalloidin to visualize actin filaments (red channel) and primary antibody histone H_2AX antibody (green channel).

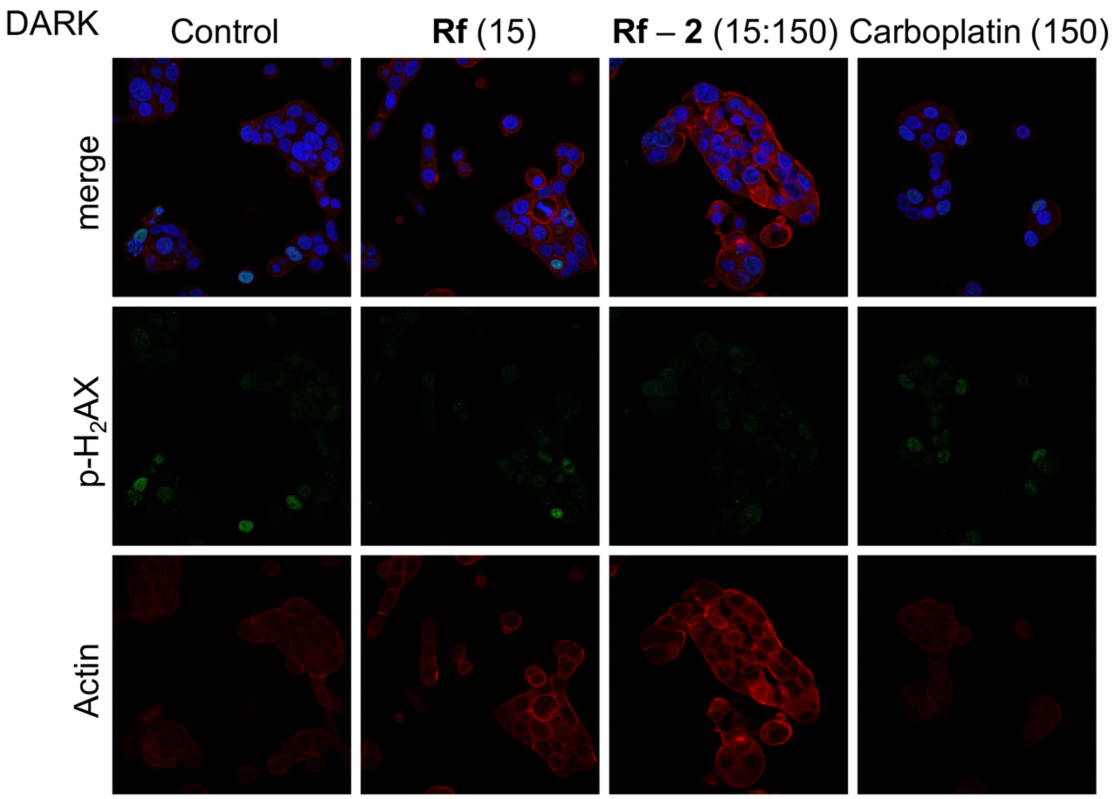

Figure S25. Immunofluorescence microscopy images of Capan-1 cells. Images correspond to control samples: **Rf** (15 µM), **Rf-2** (15–150 µM) and carboplatin (150 µM) in the dark. Cells were stained using DAPI for nuclei localization (blue channel), TRIC-phalloidin to visualize actin filaments (red channel) and primary antibody histone H_2AX antibody (green channel).

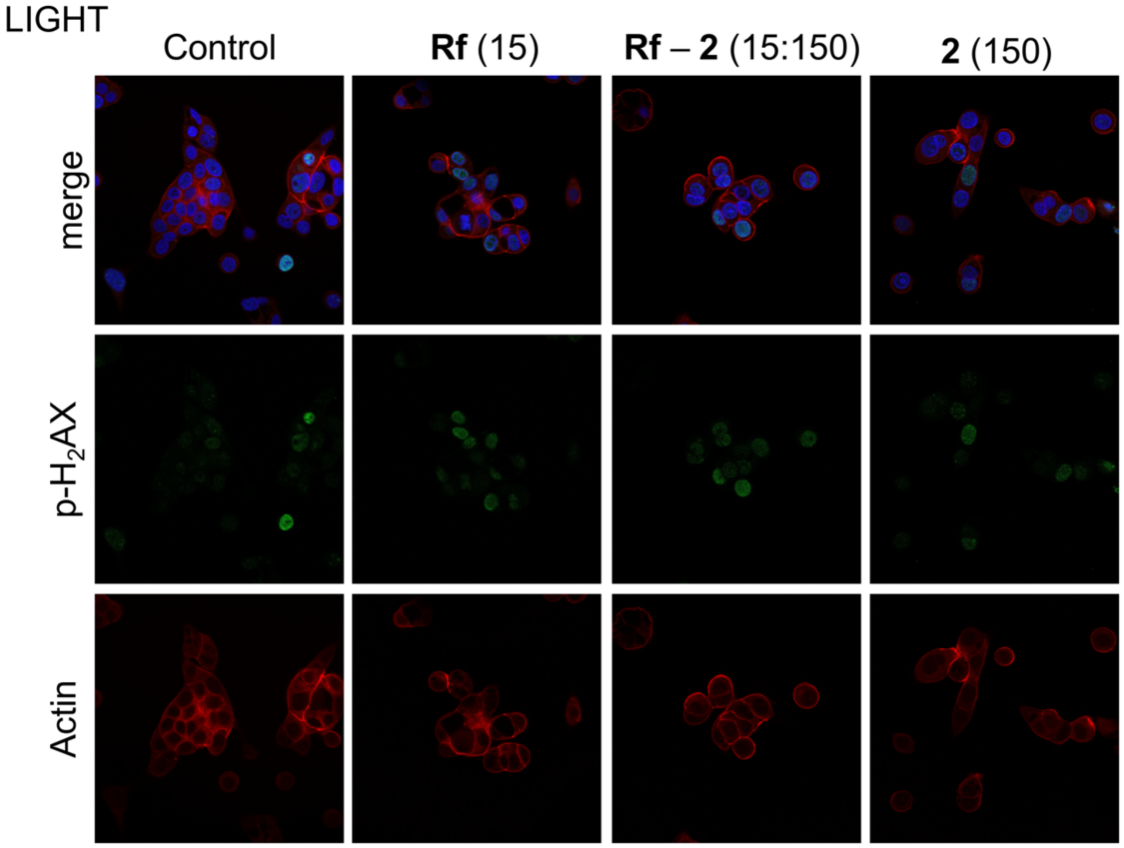

Figure S26. Immunofluorescence microscopy images of Capan-1 cells. Images correspond to control samples: **Rf** (15 µM), **Rf-2** (15–150 µM) and **2** (150 µM) irradiated 1 min at 460 nm (0.36 J·cm^{-2}). Cells were stained using DAPI for nuclei localization (blue channel), TRIC-phalloidin to visualize actin filaments (red channel) and primary antibody histone H_2AX antibody (green channel).
